# Dietary Sugar Intake and Incident Type 2 Diabetes Risk: A Systematic Review and Dose-Response Meta-Analysis of Prospective Cohort Studies

**DOI:** 10.1016/j.advnut.2025.100413

**Published:** 2025-03-21

**Authors:** Karen A Della Corte, Tyler Bosler, Cole McClure, Anette E Buyken, James D LeCheminant, Lukas Schwingshackl, Dennis Della Corte

**Affiliations:** 1 Department of Nutrition, Dietetics, and Food Science, Brigham Young University, Provo, UT, United States; 2 Institute of Nutrition, Consumption and Health, Faculty of Natural Sciences, Paderborn University, Paderborn, Germany; 3 Institute for Evidence in Medicine, Medical Center - University of Freiburg, Faculty of Medicine, University of Freiburg, Freiburg, Germany; 4 Department of Physics and Astronomy, Data Science, College of Computational, Mathematical and Physical Sciences, Brigham Young University, Provo, UT, United States

**Keywords:** dietary sugar, sugar-sweetened beverages, fruit juice, type 2 diabetes, dose-response meta-analysis

## Abstract

The dose-response relationship between dietary sugar and type 2 diabetes (T2D) risk is uncertain. MEDLINE, Embase, CINAHL, Web of Science and Cochrane databases were searched through July 9, 2024 for prospective cohort studies reporting relative measures of incident T2D risk by categories of dietary sugar (total, free, added, fructose, sucrose) or 2 beverage sources (non-diet sugar-sweetened beverages [SSBs], fruit juice) in healthy adults. Linear and restricted cubic spline dose-response models were fitted for each exposure, and study-specific slopes and confidence intervals (CIs) were calculated. Heterogeneity was evaluated using Q-statistics. Risk of bias was evaluated using the Risk of Bias in Non-randomized Studies of Exposures (ROBINS-E) tool. The Grading of Recommendations Assessment, Development and Evaluation (GRADE) approach was applied to assess the certainty of evidence. Of 10,384 studies, 29 cohorts were included: SSB: 18 (*n* = 541,288); fruit juice: 14 (*n* = 490,413); sucrose: 7 (*n* = 223,238); total sugar: 4 (*n* = 109,858); fructose: 5 (*n* = 158,136); and added sugar: 2 (*n* = 31,004). Studies were conducted in Europe (13), United States (11), Asia (6), Australia (4), and Latin America (3). Each additional serving of SSB and fruit juice was associated with a higher risk of T2D (risk ratio [RR]: 1.25; 95% CI: 1.17, 1.35 and RR: 1.05; 95% CI: >1.00, 1.11, respectively; moderate certainty). In contrast, 20 g/d intakes of total sugar and sucrose were inversely associated with T2D (RR: 0.96; 95% CI: 0.94, 0.98; low certainty; and RR: 0.95; 95% CI: 0.91, <1.00; moderate certainty, respectively). No associations were found for added sugar (RR: 0.99; 95% CI: 0.96, 1.01; low certainty) or fructose (RR: 0.98; 95% CI: 0.83, 1.15; very low certainty). These findings suggest that dietary sugar consumed as a beverage (SSB and fruit juice) is associated with incident T2D risk. The results do not support the common assumption that dietary sugar (i.e., total sugar and sucrose), irrespective of type and amount, is consistently associated with increased T2D risk.

This study was registered in PROSPERO as CRD42023401800.

**Statement of significance**

This study is the first to comprehensively establish a dose-response relationship between dietary sugar intake and type 2 diabetes risk, showing that sugar from beverages (sugar-sweetened beverages and fruit juice) increases risk, whereas total sugar, sucrose, fructose and added sugar exhibit inverse or null associations. These findings challenge the assumption that all sugars uniformly elevate type 2 diabetes risk.

## Introduction

Reducing the growing public health burden of type 2 diabetes (T2D) has become a key global health priority [1]. The intake of dietary sugars is often linked to the development of T2D, but inconsistencies remain based on the type, source, and amount of sugar [2]. Extensive pooled evidence has shown that sugar-sweetened beverages (SSBs) are adversely related to T2D risk [3,4]. Public health recommendations for reducing sugar intake predominantly stem from research focused on SSBs, given how readily measurable their consumption is and the well-documented link of SSB intake to T2D. Although this focus on SSBs is important due to their high consumption in United States diets [5] and their concentrated sugar content, it leaves a significant gap in understanding the dose-response relationship between other types of dietary sugar intake, such as total sugars, added, free sugars, sucrose, and fructose, and T2D incidence. It remains unclear whether these sugars, when consumed in various forms or at varying levels, have similar harmful relationships. Indeed, it has been reported both in prospective cohort studies and controlled feeding trials that the intake of fructose-containing sugars, independent of food form, is not linked to increased cardiometabolic disease risk [[6], [7], [8]]. The role of sugars from fruit juice compared to SSB and T2D risk has also not been adequately compared. Additionally, the potential for a threshold beyond which sugar intake becomes harmful is yet to be determined. Addressing these gaps is essential to develop more comprehensive dietary guidelines that account for the intake of a broader spectrum of dietary sugar types and their impact on T2D risk.

When undertaking a global analysis across all levels of exposure, a dose-response meta-analysis (DRM) is the preferred method and uses all incremental levels of the predictor from lowest to highest [[9], [10], [11], [12]]. DRM allows for an assessment of risk ratios (RRs) per unit of measure and is superior to other methods, such as an extreme quantile meta-analysis, which only uses a fraction of the available information from included studies. A DRM is also useful when looking for potential nonlinear associations in the dose-response over the global range [9,12]. The European Food Safety Authority reviewed the health risks of dietary sugar to inform tolerable upper intake levels [13]. Although data were available for SSBs, there was insufficient information to conduct dose-response analyses for other sugar types.

To address these data gaps and provide clearer evidence to inform dietary guidelines, we performed a robust systematic review and quantitative DRM to investigate the relationship between various types and sources of dietary sugar and T2D risk. In addition to 2 major beverage sources of dietary sugar intake (SSBs and fruit juice), we included total, free, and added sugars, as well as fructose and sucrose, as they are the most commonly assessed dietary sugar categories representing both broad classifications and specific chemical sugar types. Specifically, we examined fructose, sucrose, total, free, and added sugars as well as nondietary SSB and fruit juice in relation to incident T2D in diabetes-free healthy adults. This study may help ascertain any benefit or harm of dietary sugar, helping to provide evidence of a biological gradient based on sugar type, amount, and source.

## Methods

This systematic review was registered with the International Prospective Register of Systematic Reviews (PROSPERO identifier: CRD42023401800) and was conducted according to the Meta-analysis of Observational Studies in Epidemiology guidelines [14]. Studies were selected for the review based on the participants, intervention, comparison, outcome, and study design criteria (Supplemental Table 1).

### Search strategy and selection

We conducted a systematic literature search of MEDLINE, Web of Science, Cochrane, Embase, and CINAHL library databases on February 28, 2023 (updated July 9, 2024). Broad search terms included those related to types of dietary sugar, T2D, and noninterventional studies (see Supplemental Table 2). Surveys and reports known to the authors or found through manual searches (screening reference lists of relevant publications) were also included. We searched with no time restriction and only considered studies that were in the public domain, had summarized data, and were published in English.

After the removal of duplicates, 2 investigators independently screened articles on the basis of title and abstract from all 5 databases for potentially relevant studies (Supplemental Figure 1). The full texts of these studies were independently reviewed by 2 reviewers according to predefined inclusion and exclusion criteria (Supplemental Table 3). We considered studies to be eligible for inclusion if they were prospective cohort studies of ≥2 years in duration that assessed the intake of dietary sugars (total sugars, added sugars, free sugars, fructose, sucrose), or select food sources of sugar (nondietary SSBs, fruit juice) and ascertained incident T2D. Healthy adults aged ≥18 years from any racial or ethnic background who did not have diabetes at study initiation were included. Only studies that reported estimates of risk relations (i.e., hazard ratio, odds ratio, relative risk) of T2D along with measures of uncertainty by categories of sugar exposure were eligible. The screening and sorting process, along with the predefined exclusion criteria, are listed in Supplemental Figure 1. Discrepancies in the selection of eligible studies were resolved by consensus. Relevant study characteristics were extracted by 2 reviewers independently (Supplemental Table 4). Relative measures of T2D risk by sugar intake categories were extracted from models adjusted for body fat and energy intake at a minimum in addition to other lifestyle and dietary factors whenever such adjustments were reported. When multiple publications from a study were available, the study with more cases, more complete data, or the longest duration of follow-up was used. A single cohort was included more than once if different sugar types measured in this cohort were reported in separate studies. When reports provided incomplete information, study authors were contacted.

We selected a 12-ounce serving size for SSBs in the DRM because it represents the standard size of a single can. Coca-Cola was used as the standard for SSBs, containing 39 g of sugar per 12-ounce serving, as documented in the USDA FoodData Central database (Food ID: 2678649). For fruit juice, we chose an 8-ounce serving, as this is the recommended portion size and equivalent to 1 cup. We referenced an orange juice drink with 23.3 g of sugar per 8-ounce serving, according to the USDA FoodData Central database (Food ID: 169044). Studies reporting on fructose had varied definitions; for example, Ahmadi-Abhari et al. [15] and Meyer et al. [16] both analyzed fructose as an individual sugar. Kanehara et al. [17] computed total fructose intake as fructose intake + ½ sucrose intake. Schulze et al. [18] did not provide definitions for fructose.

### Statistical analysis

We applied a random-effects model to derive summary RRs and 95% CIs to investigate the associations of different sugar types with T2D incidence [19]. The standard error for the logarithm RR of each study was obtained via the inverse variance method and was considered the estimated variance of the logarithm RR [19]. The meta-analysis pooled all risk ratio effect measures (odds ratio, relative risk, and hazard ratio). For the dose-response analysis, we applied 2-stage random-effects meta-analyses described by Greenland and Longnecker [10] and Orsini et al. [11], as implemented in the *dosresmeta* R package. Data on cases, noncases, and person-years, along with the RRs and 95% CIs, were required for >2 quantitative exposure categories to apply the cubic spline modeling method.

Linear models and restricted cubic splines for each study were calculated to compare linear with possible nonlinear associations. We used 3 fixed knots at the 10th, 50th, and 90th percentiles of the reported intake range for the cubic spline modeling. These models were fitted separately for each study using the *dosresmeta* package in R [20] and then combined in a multivariate random-effects meta-analysis to examine the dose-response trends, which were visualized on graphs. The pooled RRs were considered statistically significant if their 95% CIs did not include the null value of 1.00. RRs and 95% CIs were rounded to 2 decimal places; when values were rounded to the significance threshold of 1.00, we indicated if the full precision value was rounded down (“>”) or up (“<”). We used a *P* value < 0.05 in all tests to determine statistical significance of tests for linearity or heterogeneity. Q test and the *I*^2^ statistic were used to quantify heterogeneity, with a value >50% for the *I*^2^ statistic indicating potentially important statistical heterogeneity [21]. For linear models, we extracted the fixed-effects coefficients and CIs from the *dosresmeta* output and used Python to interpolate the pooled relative risk at 20 g/d of sugar intake, providing a standardized reference point for interpretation. The amount 20 g/d represents a reasonable and comparable dose and falls within the upper and lower bounds of intakes measured within studies.

We assigned the median or mean intake for each quantile to the corresponding risk estimate. For open-ended lower bounds, the median was set assuming the starting point is 0 (e.g., 5 for <10). For closed ranges, the midpoint was used (e.g., 15 for 10–20). For open-ended upper bounds, the difference between the previous group’s midpoint and its lower bound was added to the start of the last group to estimate the median (e.g., 15 − 10 = 5, added to 20 to yield 25). If studies reported exposure of SSB or fruit juice in serving sizes but did not specify the amount, recommended serving sizes for SSB and fruit juice conversions were used (Supplemental Table 3).

According to the Cochrane Handbook guidelines, publication bias should be evaluated for analyses that include ≥10 studies. Therefore, funnel plots, Begg’s test, and Egger’s test were performed for SSB and fruit juice categories only [22,23] (Supplemental Figure 2).

### Risk of bias and certainty of evidence assessment

To improve the validity of the findings, potential risks of bias were assessed using the ROBINS-E (Risk of Bias in Non-randomized Studies of Exposures) tool [24,25]. This tool measures bias in the selection of participants, classification of exposures, measurement of outcomes, and control of confounding, as well as biases due to deviations from intended interventions and missing data (see Supplemental Tables 4 and 5).

The certainty of evidence for each exposure and outcome association was rated using the Grading of Recommendations Assessment, Development and Evaluation (GRADE) criteria [26,27]. GRADE rates the certainty of a body of evidence based on the following domains: within-study risk of bias, inconsistency, indirectness, and imprecision between studies, as well as publication bias, large magnitude of effect, and dose-response gradient. Each pooled estimate was assessed using these criteria to rate the confidence for each dietary variable-disease outcome. Included observational studies started at high-quality evidence because of the additional use of ROBINS-E tool and were then downgraded due to weaknesses inherent in the observational design as well as other prespecified criteria [28]. Criteria to downgrade included study limitations (weight of studies showed risk of bias by ROBINS-E), inconsistency (substantial unexplained interstudy heterogeneity, *I*^2^ > 50% and *P* < 0.10), indirectness (presence of factors relating to the population, exposures, and outcomes that limit generalizability), imprecision (95% CIs were wide or crossed the minimally important threshold, 1.0) and publication bias (significant evidence of small-study effects). Criteria for upgrading included a large effect size (RR > 2 or RR < 0.5). Upgrading for a dose-response gradient was not applied due to the risk of residual confounding [29].

## Results

A total of 10,384 records were retrieved from the databases. Through screening and application of inclusion and exclusion criteria, 321 articles were identified for full-text review, of which 239 were duplicates (see Supplemental Figure 1). Of the 106 studies reviewed in full, 82 were excluded (see Supplemental Table 3 for full list of exclusions). Five additional studies were found via manual search [18,[30], [31], [32], [33]]. Finally, we identified 29 prospective cohort studies that were included in the final analysis [[15], [16], [17], [18],^31^,^32^,[34], [35], [36], [37], [38], [39], [40], [41], [42], [43], [44], [45], [46], [47], [48], [49], [50], [51], [52], [53], [54], [55], [56]]. The 29 reports were of 25 cohort studies involving 801,530 unique participants.

The main characteristics of the selected studies are presented in Supplemental Table 6. Six studies were conducted in Asia, 4 in Australia, 13 in Europe, 3 in Latin America, and 11 in the United States. The follow-up period ranged from 2.2 to 24.0 y, and mean follow-up duration was 12.4 y for SSB, 12.5 y for fruit juice, 8.9 y for sucrose, 8.8 y for total sugars, 7.6 y for fructose, and 7.7 y for added sugars. Dietary assessment of 22 studies was performed using validated food frequency questionnaires; 7 studies used either a diet record, food diary, diet history, or a dietary interview. All studies with multivariable models were adjusted for potential confounders. The prespecified primary confounding variable was age, for which all studies adjusted. Out of the 29 included studies, most controlled for BMI (*n*  =  28), energy intake (*n*  =  25), physical activity (*n*  =  27), smoking (*n* = 28), alcohol drinking (*n*  =  24), education level (*n* = 20), and family history of diabetes (*n*  =  18). Ascertainment of incident cases was objectively performed by most studies either using a medical record (76%), a biomarker assessment to validate diagnosis (17%), or by confirmed use of T2D medication (7%). Six studies reported results for men and women separately [17,18,32,36,39,49], 3 cohorts only included men [36,44,53], and 9 cohorts only included women [16,31,[34], [35], [36],^40^,^42^,^48^,^51^], and 1 study reported results for 2 cohorts separately [36]. No study on free sugars as a sugar predictor were found. Supplemental Table 7 presents specific data on outcomes and sugar intake amounts, and Supplemental Figure 3A-G shows linear and cubic spline fits for all sugar categories by individual study as well as aggregated.

### SSB results

**FIGURE 1:**
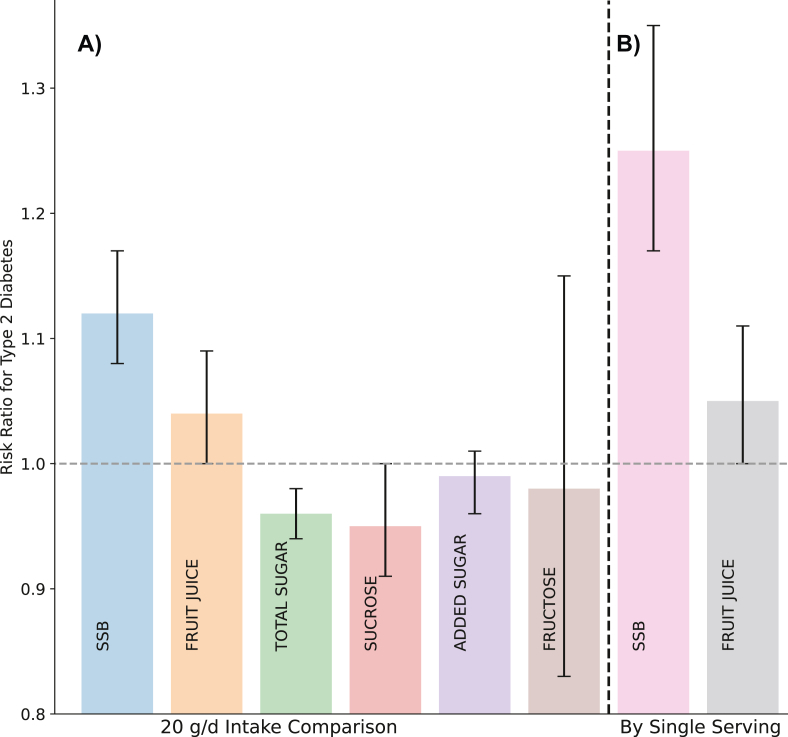
Overall pooled effect of 20 g/d sugar intake on T2D risk: comparison of sugar categories and typical doses of SSB and fruit juice. (A) Bar plot illustrating the summary effect of a 20 g/d intake for different sugar types on risk of T2D. (B) The rightmost bars compare these doses to typical servings sizes per day of SSBs (39 g/d) and fruit juice (23.3 g/d). SSB, sugar-sweetened beverage; T2D, type 2 diabetes.

**FIGURE 2:**
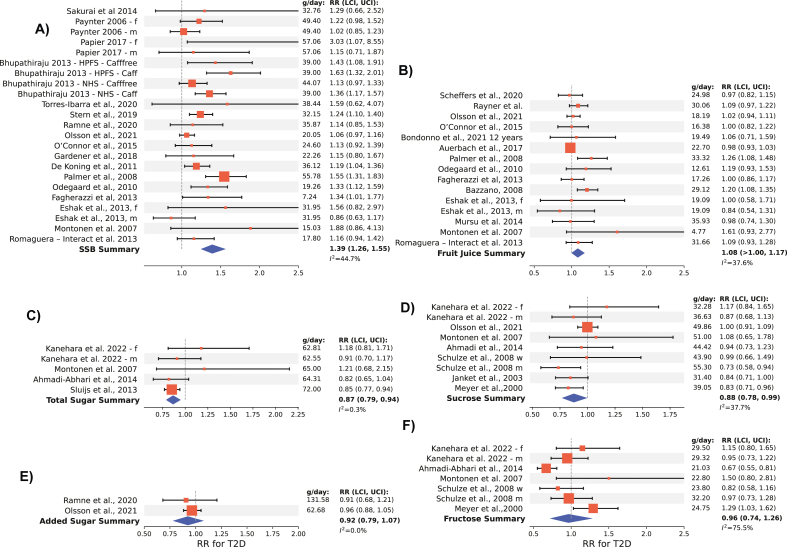
Forest plots of risk ratios for extreme quantiles of daily sugar consumption by sugar category and the risk of T2D. Summary line represents the overall relative risk comparing the highest intake category to the lowest intake category as reported in the studies, with lower and upper 95% confidence intervals in parenthesis. Grams/day represent the highest intake dose in each study. Subplots of T2D risk for the following sugar categories: (A) SSBs, (B) fruit juice, (C) total sugar, (D) sucrose, (E) added sugar, and (F) fructose. Caff, caffeinated; Cafffree, caffeine-free; f, female; HPFS, Health Professionals Follow-Up Study; LCI, lower confidence interval; m, male; NHS, Nurses’ Health Study; RR, risk ratio; SSB, sugar-sweetened beverage; T2D, type 2 diabetes; UCI, upper confidence interval.

**FIGURE 3:**
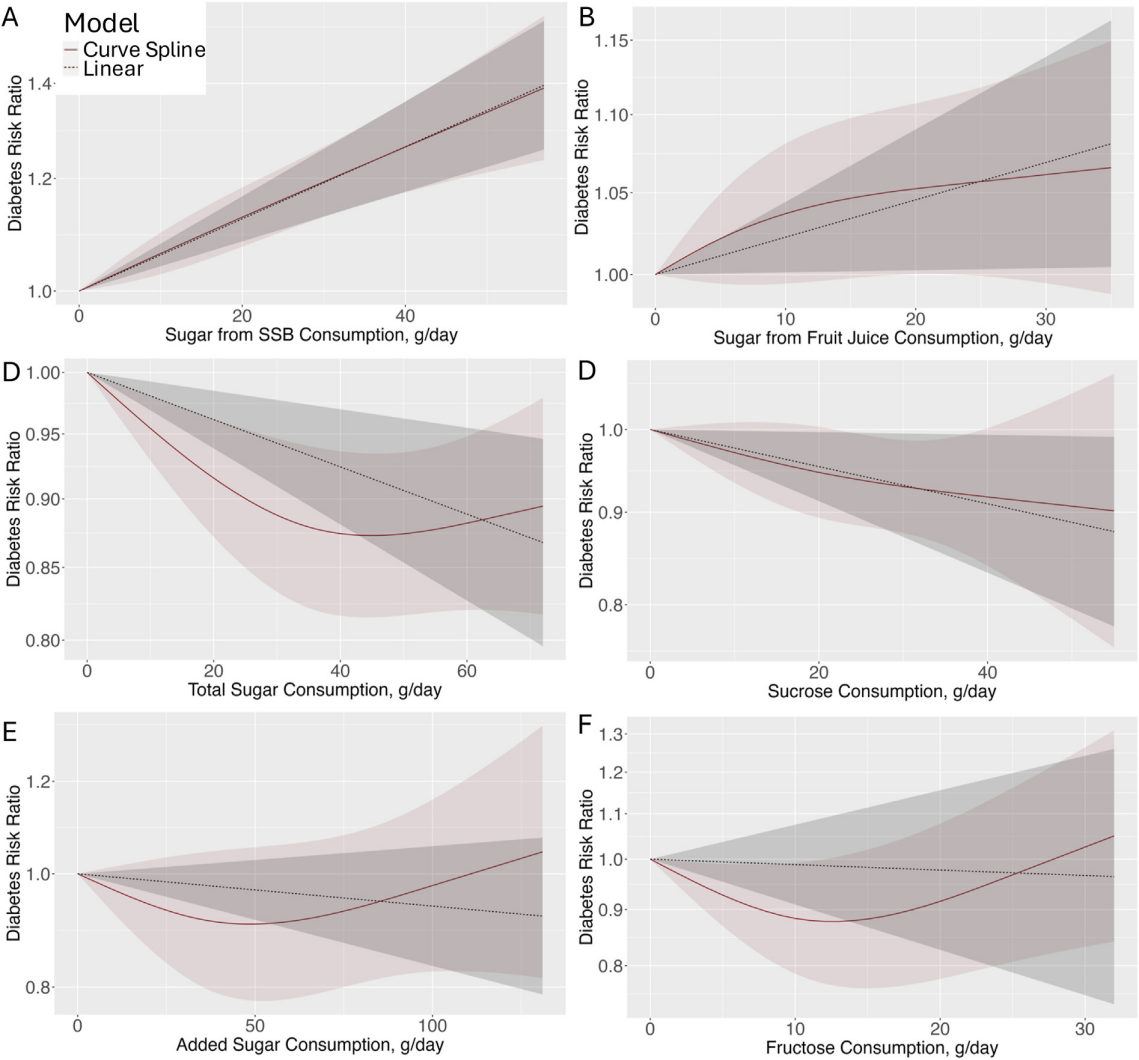
Dose-response relationship between incident T2D risk and consumption of (A) sugar from SSB, (B) sugar from fruit juice, (C) total sugars, (D) dietary sucrose, (E) added sugars, and (F) dietary fructose. Panels depict dose-response relationships modeled using linear and nonlinear approaches. Shaded regions represent 95% confidence intervals. Red shaded regions correspond to the nonlinear model fit, using restricted cubic splines to capture potential nonlinearity in the relationship. Grey shaded regions represent the linear model fit, assuming a constant rate of change across all dose levels. Solid lines show the point estimates for each mode: solid red line reflects the nonlinear model; dashed grey line corresponds to the linear model. The x-axis represents maximum intake ranges across all included studies for a given sugar type, and the y-axis shows relative risk of T2D. SSB, sugar-sweetened beverage; T2D, type 2 diabetes.

Seventeen studies (*n* = 541,288; 43,532 cases) were included to analyze the dose-response relationship between SSB and incident T2D risk. With each additional serving of SSB per day, the risk of developing T2D increased by 25% (RR: 1.25; 95% CI: 1.17, 1.35; *I*^2^ = 45%; moderate certainty) (Figure 1; Supplemental Table 8). Comparing categories of highest compared with lowest intakes of SSB, a significant association with the risk of T2D was observed (RR: 1.39; 95% CI: 1.26, 1.55; *P*-heterogeneity = 0.011; *n* = 23) (Figure 2). Compared to other dietary sugar categories, a 20 g/d intake of sugar from SSB was associated with the greatest increase in risk of T2D (RR: 1.12; 95% CI: 1.08, 1.17), and data were consistent with a dose-response linear relationship (FIGURE 1, FIGURE 3; *P*-nonlinearity = 0.87).

### Fruit juice results

Fourteen studies (*n* = 490,413; 43,065 cases) were included in the DRM for fruit juice and incident T2D risk. With each additional serving of sugar from fruit juice per day, the risk of developing T2D increased by 14% (RR: 1.05; 95% CI: >1.00, 1.11; moderate certainty) (Figure 1; Supplemental Table 8). Comparing categories of highest compared with lowest intakes of sugar from fruit juice, we observed an association with the risk of T2D (RR: 1.08; 95% CI: >1.00, 1.17; *I*^2^ = 38%; *P*-heterogeneity = 0.070; n = 15) (Figure 2). Compared to other dietary sugar categories, a 20 g/d intake of sugar from fruit juice was associated with a 4% increase in T2D risk (RR: 1.04; 95% CI: >1.00, 1.09) and followed a linear dose-response relationship (*P*-nonlinearity = 0.34) (FIGURE 1, FIGURE 3).

### Total sugar results

Four studies (*n* = 109,858; 13,675 cases) were included to meta-analyze the dose-response relationship between total sugar intake and incident T2D risk. The intake of an additional 20 g/d of total sugar was associated with a significant reduction in T2D risk (RR: 0.96; 95% CI: 0.94, 0.98; low certainty) (Figure 1; Supplemental Table 8). Comparing categories of highest compared with lowest intakes of total sugar, a decrease in T2D risk was observed (RR: 0.87; 95% CI: 0.79, 0.94; *I*^2^ = 0.3%; *P*-heterogeneity = 0.404; *n* = 5) (Figure 2). Evidence of a nonlinear dose-response association was detected (*P*-nonlinearity = 0.026) (Figure 3). T2D risk decreased with total sugar intake up to ∼40 to 60 g/d, after which the risk plateaued and slightly increased, although the pooled RR remained <1.0, showing a continued protective association.

### Dietary sucrose results

Seven studies (*n* = 223,238; 9065 cases) were included to meta-analyze the dose-response for dietary sucrose intake and incident T2D risk. An increase in sucrose intake by 20 g/d was associated with a reduction in risk of T2D (RR: 0.95; 95% CI: 0.91, <1.00; moderate certainty) (Figure 1; Supplemental Table 8). Comparing categories of highest compared with lowest intakes of dietary sucrose, a decrease in risk was observed (RR: 0.88; 95% CI: 0.78, 0.99; *I*^2^ = 38%; *P*-heterogeneity = 0.118; *n* = 9) (Figure 2). Data were consistent with a linear dose-response relationship (*P*-nonlinearity = 0.720) (Figure 3).

### Added sugar results

Two studies (*n* = 31,004; 4796 cases) were included to analyze the dose-response relationship for added sugar intake and incident T2D risk. An increase in added sugar intake by 20 g/d was not associated with the risk of T2D (RR: 0.99; 95% CI: 0.96, 1.01; low certainty) (Figure 1; Supplemental Table 8). Comparing categories of highest and lowest intakes of added sugar, no association with the risk of T2D was observed (RR: 0.92; 95% CI: 0.79, 1.07; *I*^2^ = 0.0%; *P*-heterogeneity = 0.909; *n* = 2) (Figure 2). No evidence of a nonlinear dose-response association was detected (*P*-nonlinearity = 0.180), however a slight J-shaped relationship was observed (Figure 3).

### Dietary fructose results

Five studies (*n* = 158,136; 4101 cases) were included in the DRM for dietary fructose intake and incident T2D risk. An increase in fructose intake by 20 g/d was not associated with the risk of T2D (RR: 0.98; 95% CI: 0.83, 1.23; very low certainty) (Figure 1; Supplemental Table 8), with evidence of substantial heterogeneity (*I*^2^ = 76%; *P*-heterogeneity ≤ 0.001); *n* = 7). Comparing categories of highest compared with lowest intakes of dietary sucrose, no association with T2D risk was observed (RR: 0.97; 95% CI: 0.74, 1.26) (Figure 2). Evidence of a nonlinear dose-response association was detected (*P*-nonlinearity = 0.002). T2D risk decreased with increasing fructose intake up to ∼10 to 15 g/d, after which risk began to increase (Figure 3).

### Risk of bias

No study was judged as having low risk of bias due to the nonrandomized observational nature of the studies, the possibility of residual confounding, and the possibility of measurement errors in dietary assessments. Thus, all publications were judged as having a moderate risk of bias except for 1 [55], which was judged as having a serious risk of bias due to its nested case-cohort study design (Supplemental Table 5). Supplemental Table 6 lists the ROBINS-E description and decision criteria for each risk of bias domain. The results of the risk of bias were considered as part of the GRADE evaluation (for full details of the GRADE evidence, see Supplemental Table 8).

### Sensitivity analysis and publication bias assessment

For all dietary sugar exposure categories, the significance or direction of the association was not altered by the removal of any single study at a time (Supplemental Table 9).

Visual inspection of funnel plots and formal statistical tests indicated no evidence of publication bias for studies on SSBs or fruit juice. For SSBs, Begg’s test (Kendall’s tau = 0.028, *P* = 0.876) and Egger’s test (t = 0.760, df = 21, *P* = 0.456) were nonsignificant, with a limit estimate of 0.066 (95% CI: 0.01, 0.12) as the standard error approached 0. Similarly, for fruit juice, Begg’s test (Kendall's tau = −0.143, *P* = 0.495) and Egger’s test (t = −0.100, df = 13, *P* = 0.922) were nonsignificant, with a limit estimate of 0.028 (95% CI: −0.07, 0.13). These findings suggest no significant asymmetry in the funnel plots, indicating an absence of publication bias for studies on both beverages.

## Discussion

In this systematic review and meta-analysis of prospective cohort studies on the dose-response relationship between dietary sugar intake (total, added, sucrose, fructose, SSB, and fruit juice) and T2D incidence, we found that risk was influenced by the form in which sugar was consumed. Specifically, each additional serving of SSBs and fruit juice increased the risk of T2D by 25% and 5%, respectively. In contrast, the intakes of sucrose and total sugar (which includes any consumption of natural sugars as well as added) were inversely associated with T2D, indicating a protective relationship. However, added sugar and fructose did not show clear associations with T2D risk.

Consensus is lacking whether the fructose component of sugar and high-fructose corn syrup confers a greater risk or if sugar intake is simply a vehicle for increased caloric intake. Systematic reviews and meta-analyses of controlled feeding trials have demonstrated that the detrimental effects of sugars on weight gain and cardiometabolic risk factors are primarily mediated by excess energy intake, with evidence of harm reported in studies where sugars were added to diet as excess energy [[57], [58], [59], [60], [61]]. However, in our study, the harmful associations of sugars from SSBs and fruit juice on T2D risk were reported even with adjustment for energy intake and BMI in all but 4 studies. This suggests that the risk of dietary sugar consumed in liquid form may stem from adverse metabolic effects beyond excess calories and body weight. Evidence from intervention studies has shown that liquid fructose is a potent stimulator of hepatic de novo lipogenesis (DNL) [62,63] and worsens insulin sensitivity in both hyper- and isocaloric settings [64,65]. For example, Stanhope et al. [64] reported that consuming fructose-sweetened beverages (25% of daily energy) for 10 wk significantly increased DNL, fasting insulin, and blood glucose and decreased insulin sensitivity, effects not observed with glucose [58]. Similar effects have been reported at lower doses: a 12-wk study found that consuming 75 g/d of liquid fructose raised insulin levels and HOMA-IR in obese men compared to baseline [62], whereas a 3-wk study providing 80 g/d showed that fructose increased endogenous glucose production and impaired hepatic glucose suppression, effects not seen with equivalent glucose intake [66]. These findings highlight that fructose-containing SSBs uniquely disrupt liver metabolism and elevate insulin resistance. In our study, fructose was mainly measured as an individual sugar, not part of sucrose or high-fructose corn syrup, and had high inconsistency in the results.

Compared to sugars from SSBs, which provide empty calories, fruit juice can contain beneficial nutrients such as vitamins and phytochemicals; however, our study found that sugar consumption from fruit juice was positively associated with T2D risk. The high sugar content and lack of fiber in fruit juice are similar to SSBs, making it a poor substitute for whole fruits, which provide higher fiber content to support better blood glucose regulation. SSBs supply isolated sugars leading to a greater glycemic impact, whereas other sources of dietary sugars, particularly when consumed in nutrient-dense foods such as whole fruits, dairy products, or whole grains, may elicit slower blood glucose responses due to accompanying fiber, fats, or proteins.

Other sources of fructose-containing sugars, such as those added to grain products, yogurt, fruit, and dairy, contribute significantly to total sugar intake, which includes both naturally occurring and added sugars. We observed a significant inverse association between total sugars and T2D incidence in our study. The beneficial impact of total sugars on glucose metabolism may be explained by the favorable effects of micronutrients and bioactive polyphenols found within natural sources of sugars and the fact that many of these foods have a lower glycemic index (e.g., whole fruit, whole grain, milk and dairy products) [67]. A high intake of fruits, which are naturally high in fructose, is associated with good metabolic health, and when consumed in whole form rather than as juice is inversely associated with T2D risk [35,[68], [69], [70], [71]]. Additionally, nutritious foods with sugar added to them such as whole-grain cereals and yogurt have been associated with a reduced risk of T2D [72], possibly due to their nutrient and phytochemical profiles. Indeed, the inclusion of sugar in these healthful foods may enhance their consumption, which could explain why dietary sucrose, a common form of added sugar, was inversely associated with T2D in our study. Thus, we posit that the food source in which sugars are consumed needs to be considered when evaluating the complex relationship between dietary sugar and disease outcomes [73].

Although fructose is not consumed alone to any appreciable degree in human diet, a great deal of research has focused on this monosaccharide. In 2011, the European Food Safety Authority issued a scientific opinion on fructose, stating that “consumption of fructose leads to a lower blood glucose rise than consumption of sucrose or glucose,” although noting that high intakes of fructose (set at >25% of total energy) were shown to lead to metabolic complications such as dyslipidemia, insulin resistance, and increased visceral adiposity [74]. This dose-dependent relationship, along with the various definitions of fructose (individual or including 50% of sucrose) in our studies may explain the high degree of variation observed in our fructose meta-analysis, which is why it was graded as very low in its certainty of evidence.

Several scientific organizations, including the WHO, the Scientific Advisory Council on Nutrition, and the American Heart Association, have recommended significant restrictions on upper limits of sugars consumption [[75], [76], [77]]. The WHO recommended that <10% of energy be derived from free sugars based primarily on evidence of detrimental effects of SSB, particularly on weight status [78]. However, there was no definitive science indicating that 10% is a threshold for increased risk. Instead, the committee performed modeling that suggested that diets providing 4% to 6% of energy from added sugars could still meet recommended nutrient levels and stay within total energy limits for diets ranging from 1200 to 2800 kcal/d [79]. Americans consume ∼13% of energy from added sugars [80], making the 10% threshold more of a reduction goal than an optimal intake level.

Our DRM aligns with these guidelines to some extent but also highlights important nuances. Our findings indicate that the risk of T2D increases in a dose-response fashion at all levels of sugar intake from liquid sources, with no apparent upper tolerable intake level for these sources. This underscores the need for even more stringent recommendations for liquid sugars such as those in SSBs and fruit juice, as they appear to harmfully associate with metabolic health. Rather than condemning all added sugars, future guidelines might consider the differential effects of sugar based on its source and form. Dietary sugar in the context of nutrient-dense foods may have a different risk profile than liquid sources, underscoring the importance of assessing sugar intake within the broader dietary matrix.

### Strengths and limitations

Nutrition-related meta-analyses of prospective studies offer insights based on real-world dietary intakes in natural settings, enhancing the generalizability of their findings. A follow-up study design is often required for the investigation of hard disease endpoints such as T2D incidence, which may take years to develop. Thus, when studying the relationship between dietary sugar and the development of T2D, prospective cohort studies represent the strongest evidence available among epidemiological approaches. A previous (2017) systematic review and meta-analysis on non-SSB sugars and T2D incidence in observational studies compared upper and lower quantiles of intake and found no adverse associations between fructose-containing sugars and incident T2D, and sucrose intake was associated with a decreased risk [81]. Compared to this review, our systematic review and meta-analysis applied a dose-response meta-regression at all levels of intake from lowest to highest [[9], [10], [11], [12]]. Other meta-analyses associating incident T2D with sugary beverages used extreme quantile comparisons. A further strength is that we performed an extensive systematic search of 5 databases and investigated SSB and fruit juice as well as 5 other sugar categories for a comprehensive overview of various sugar types and T2D risk. Finally, the removal of individual studies during sensitivity analyses did not impact the direction or significance of all results, indicating the robustness of the findings.

Despite the inclusion of large, high-quality cohorts, the inability to rule out residual confounding is a limitation inherent in all observational studies. High consumers of SSBs tend to have a higher overall energy intake, engage in less physical activity, and have higher rates of smoking [[82], [83], [84]]. Although almost all included studies controlled for these lifestyle factors and used validated dietary assessment methods, residual confounding could include unmeasured dietary and lifestyle factors as well as the reliability of self-reported intake [85], which could affect the results of the dose-response analyses [86]. Evidence for inconsistency was seen in the dietary fructose analysis, which had wide CIs preventing any clear conclusions to be drawn about a clinically important benefit or harm of this sugar, especially because definitions of fructose diverged between included studies. A further limitation is that we did not include specific sugar sources such as candy, ice cream, or various syrups in the meta-analysis. This decision was made to focus on the broader sugar categories and specific chemical types as well as the 2 most frequently consumed beverage sources to ensure a globally comprehensive yet focused synthesis of the most commonly studied sugars. A final limitation is the imprecision in the estimates of pooled risk for added sugars and fructose, which contributed to downgrades in GRADE assessments for these exposures. The evidence for total sugars was rated as low quality due to the inclusion of 1 study with a high-risk of bias. Progressive dose-response gradients—positive for SSB and fruit juice and negative for total sugar and sucrose intake—were observed; however, we did not upgrade for these as is typically done in GRADE due to the risk of residual confounding.

## Conclusion

In this systematic review and meta-analysis of prospective cohort studies on the dose-response relationship between dietary sugar intake and T2D incidence, we found that SSBs and fruit juice were associated with an increased risk of T2D (moderate certainty), while intakes of total sugar (low certainty) and sucrose (moderate certainty) were inversely associated with T2D risk, and no clear relationships were observed for added sugar and fructose. Our findings suggest the importance of sugar type in determining the association of dietary sugar, with higher liquid sugar intakes apparently linked to greater harm. Our results do not support the common assumption that dietary sugar intake, irrespective of type and amount, is consistently associated with an increased risk of T2D. Future research is needed to evaluate the long-term impacts of reducing liquid sugar consumption on T2D prevention and to investigate the mechanisms underlying the differing effects of liquid and solid sugars.

## Author contributions

The authors’ responsibilities were as follows – KDC: conceived and designed the research; TB, CL, KDC: conducted the systematic search independently; TB, CL: extracted data from each report; TB, DDC: prepared the data for analysis; TB: prepared the tables; DDC: conducted the meta-analysis and prepared the figures with KDC; LS: provided consultation in conducting the meta-analysis and quality of evidence; AEB: provided subject matter expertise in critically reviewing the manuscript; JDL: provided support and training to the students TB and CL; KDC, DDC: conducted risk of bias and GRADE analyses; KDC: interpreted the results and wrote the manuscript; KDC: had primary responsibility for the content of the manuscript; and all authors: read and approved the final manuscript.

## Data availability

Data described in the manuscript, code book, and analytic code will be made available upon request pending application and approval.

## Funding

The authors reported no funding received for this study.

## Conflict of interest

The authors declare the following financial interests/personal relationships which may be considered as potential competing interests: LS is an associate editor of *Advances in Nutrition*. AEB reports a relationship with German Research Foundation that includes: funding grants. AEB reports a relationship with European Commission Seventh Framework Programme for Research and Technological Development Joint Technology Initiatives that includes: funding grants. AEB reports a relationship with Nutrition that includes: board membership. AEB reports a relationship with German Society for Nutrition eV that includes: speaking and lecture fees. AEB reports a relationship with Chinese GI Foundation that includes: speaking and lecture fees. AEB reports a relationship with German Society for Pediatric and Adolescent Medicine eV that includes: speaking and lecture fees. AEB reports a relationship with International Life Sciences Institute Europe ILSI that includes: speaking and lecture fees. AEB reports a relationship with Ernahrung im Fokus that includes: speaking and lecture fees. AEB reports a relationship with Paderborn University Head Ethics Committee that includes: board membership. AEB reports a relationship with Nutriscore that includes: board membership. All other authors report no conflicts of interest.
