# Supplementary material for: Dietary Sugar Intake and Incident Type 2 Diabetes Risk: A Systematic Review and Dose-Response Meta-Analysis of Prospective Cohort Studies
Source: Adv Nutr. 2025 Mar 21;16(5):100413. doi: 10.1016/j.advnut.2025.100413 (PMC12145082; doi:10.1016/j.advnut.2025.100413)
Supplement: multimedia component 1 [file mmc1.docx]

*Supplementary Material*

**Dietary Sugar Intake and Incident Type 2 Diabetes Risk: A Systematic Review and Dose-Response Meta-Analysis of Prospective Cohort Studies**

Karen A. Della Corte ([karen_dellacorte@byu.edu](mailto:karen_dellacorte@byu.edu))

**Table of Contents**

[Supplementary Table 1 Eligibility criteria by the PICOS statement 2](#_Toc192494413)

[Supplementary Table 2 Literature Search Strategy for MEDLINE, EMBASE, CINAHL, Web of Science and COCHRANE Databases 3](#_Toc192494414)

[Supplementary Figure 1 Sorting diagram by exclusion criteria for systematic search of five databases 4](#_Toc192494415)

[Supplementary Table 3 Studies reviewed in full text for eligibility and included or excluded for meta-analysis 5](#_Toc192494416)

[Supplementary Figures 2 A-B Risk of publication bias analysis 9](#_Toc192494417)

[Supplementary Table 4 Risk of bias judgements for each domain (from the ROBINS-E detailed guidance) 10](#_Toc192494418)

[Supplementary Table 5 Description and decision criteria for each domain in ROBINS-E 11](#_Toc192494419)

[Supplementary Table 6 Study characteristics 15](#_Toc192494420)

[Supplementary Table 7 A-G Outcome statistical data and intake amounts by sugar category 21](#_Toc192494421)

[Supplementary Table 8 Grade assessment for dose-response analysis of dietary sugar exposures and T2D incidence 28](#_Toc192494422)

[Supplementary Table 9 A-G Results from leave-one-out sensitivity analyses for each sugar category. See Table S6 for references of studies. 29](#_Toc192494423)

[9-A Total Sugar 29](#_Toc192494424)

[9-B Total Sucrose 29](#_Toc192494425)

[9-C Added Sugar 29](#_Toc192494426)

[9-D SSB 30](#_Toc192494427)

[9-E Fruit Juice 30](#_Toc192494428)

[9-F Fructose 31](#_Toc192494429)

[9-G Glucose 32](#_Toc192494430)

[Supplementary Figures 3 A-G Linear and cubic spline fits for all sugar types by study level and aggregated. 33](#_Toc192494431)

[References 35](#_Toc192494432)

# **Supplementary Table 1** Eligibility criteria by the PICOS statement

|  | Inclusion criteria | Exclusion criteria |
| --- | --- | --- |
| P (population) | Generally healthy adult population from any racial or ethnic background. | Specific patient populations, studies on children, adolescents or pregnant women |
| I (intervention/exposure) | Dietary sugar intake (e.g. SSB, fruit juice, total sugars, added sugars, free sugars, sucrose, fructose and glucose). | Studies that do not provide clear, quantifiable measures of dietary sugar intake. Non-dietary sources of sugar (e.g., intravenous administration). Studies that assess sugar intake as part of a broader dietary pattern without isolating the specific impact of sugar. |
| C (comparison) | Cohorts where T2D risk ratios of varying levels of sugar intake (e.g., quantiles of intake) are compared. | Studies that do not differentiate between types or levels of sugar intake. Comparisons where dietary sugar intake is not quantified or categorized by T2D risk ratios. |
| O (outcome) | Type 2 diabetes incidence | Biomarkers of diabetes risk (e.g. fasting glucose without diabetes diagnosis). Studies that do not report the number of incident cases of T2D. |
| S (study design) | Prospective observational studies with at least 2 years of follow-up. | In vitro/animal experiments, cross-sectional and retrospective case-control studies |

# **Supplementary Table 2** Literature Search Strategy for MEDLINE, EMBASE, CINAHL, Web of Science and COCHRANE Databases

| MEDLINE | EMBASE | CINAHL | WEB OF SCIENCE | COCHRANE |
| --- | --- | --- | --- | --- |
| 1. Dietary Sugars[Mesh]  2. diet* fructose  3. diet* sucrose  4. Dietary Sucrose[Mesh]  5. sugar* (intake* or consum* or diet*)  6. sugar* sweetened beverage*  7. SSB (intake* or consum*)  8. soft drink* (intake* or consum*)  9. candy (intake* or consum*)  10. fruit juice* (intake or consum*)  11. sweets (intake* or consum*)  12. “food group*”  13. Diabetes Mellitus, Type 2 [Mesh]  14. Type 2 Diabetes[tiab] or T2D[tiab] or T2DM[tiab]  15. #1 or #2 or #3 or #4 or #5 or #6 or #7 or #8 or #9 or #10 or #11 or #12  16. #13 or #14  17. #15 and #16  18. #17 not (mice[tiab] or rats[tiab])  19. #18 not (trial[ti] or randomized[ti]) | 1. diet* near/10 sugar*  2. diet* near/10 fructose  3. diet* near/10 sucrose  4. sugar* near/10 intake*  5. sugar* near/10 consum*  6. sugar* sweetened beverage*  7. SSB (intake* or consum*)  8. soft drink* (intake* or consum*)  9. candy (intake* or consum*)  10. fruit juice* (intake* or consum*)  11. sweets (intake* or consum*)  12. “food group*”  13. type 2 diabetes  14. T2D*:ab,ti  15. #1 or #2 or #3 or #4 or #5 or #6 or #7 or #8 or #9 or #10 or #11 or #12  16. #13 or #14  17. #15 and #16  18. #17 not (mice:ab,ti or rats:ab,ti)  19. #18 not (‘trial’:ti or ‘randomized’:ti) | 1. diet* sugar*  2. diet* fructose  3. diet* sucrose  4. sugar* intake*  5. sugar* consum*  6. sugar* sweetened beverage*  7. SSB (intake* or consum*)  8. soft drink* (intake* or consum*)  9. candy (intake* or consum*)  10. fruit juice* (intake* or consum*)  11. sweets (intake* or consum*)  12. “food group*”  13. type 2 diabetes  14. AB T2D* or TI T2D*  15. S1 or S2 or S3 or S4 or S5 or S6 or S7 or S8 or S9 or S10 or S11 or S12  16. S13 or S14  17. S15 and S16  18. S17 not (TI randomized or TI trial)  19. S18 not (TI mice or AB mice or TI rats or AB rats) | 1. ALL=(diet* sugar)  2. ALL=(diet* fructose)  3. ALL=(diet* sucrose)  4. ALL=(sugar* intake*)  5. ALL=(sugar* consum*)  6. ALL=(sugar* sweetened beverage*)  7. ALL=(SSB (intake* or consum*))  8. ALL=(soft drink* (intake* or consum*))  9. ALL=(candy (intake* or consum*))  10. ALL=(fruit juice* (intake* or consum*))  11. ALL=(sweets (intake* or consum*))  12. ALL=“food group*”  13. ALL= type 2 diabetes  14. AB=(T2D*) or TI=(T2D*)  15. #1 or #2 or #3 or #4 or #5 or #6 or #7 or #8 or #9 or #10 or #11 or #12  16. #13 or #14  17. #15 and #16  18. #17 not TI=(randomized or trial)  19. #18 not TI=(mice or rats)  20. #19 not AB=(mice or rats) | 1. MeSH: Dietary Sugars  2. diet* fructose  3. diet* sucrose  4. sugar* intake*  5. sugar* consum*  6. sugar* sweetened beverage*  7. SSB (intake* or consum*)  8. soft drink* (intake* or consum*)  9. candy (intake* or consum*)  10. fruit juice* (intake* or consum*)  11. sweets (intake* or consum*)  12. “food group*”  13. MeSH: Diabetes Mellitus, Type 2  14. Type 2 Diabetes:ti,ab or T2D*:ti,ab  15. #1 or #2 or #3 or #4 or #5 or #6 or #7 or #8 or #9 or #10 or #11 or #12  16. #13 or #14  17. #15 and #16  18. #17 not (randomized:ti or trial:ti)  19. #18 not (mice:ti,ab or rats:ti,ab) |
| Feb 28, 2023: 2,220  Update July 9, 2024: 268 | Feb 28, 2023: 2,558  Update July 9, 2024: 401 | Feb 28, 2023: 799  Update July 9, 2024: 49 | Feb 28, 2023: 2,412  Update July 9, 2024: 404 | Feb 28, 2023: 1,124  Update July 9, 2024: 149 |

# **Supplementary Figure 1** Sorting diagram by exclusion criteria for systematic search of five databases

The systematic search and sorting process based on exclusion criteria to identify eligible studies on sugar-T2D risk relations. Medline, Embase, Web of Science, CINAHL and Cochrane databases were searched through Feb 15, 2023 (update July 9, 2024). Sugar not reported: defined as sugar not reported in quantiles of T2D risk. Figure created with PRISMA2020 (1).

# **Supplementary Table 3** Studies reviewed in full text for eligibility and included or excluded for meta-analysis

| Cohort, country | Results from full text review |
| --- | --- |
| **Identified as potentially eligible – decision to exclude** |  |
| Alcada et al. (2) | Duplicate of Palmer et al. |
| Ashton et al. (3) | Sugar not reported separately, only as a diabetes prevention index. |
| Barclay et al. (4) | Only one dose provided for analysis |
| Barrio-Lopez et al. (5) | T2D incidence not reported. |
| Bauer et al. (6) | Sugar reported as a dietary pattern score. |
| Bitton et al. (7) | Appears to be duplicate data from Palmer et al. Study not findable. |
| Den Braver et al. (8) | Sugar reported as part of dietary pattern score. |
| Chen et al. (9) | Only serum fructose levels measured and not intake amounts. |
| Colditz et al. (10) | Eligible, but intake amounts and risk relations were not reported. Authors were contacted and they responded but could not provide any information as this study is older. |
| Conklin et al. (11) | Sugar only reported as part of a dietary diversity score. |
| Den Biggelaar et al. (12) | Eligible, but not included because study is a cross-sectional. |
| Den Biggelaar et al. (13) | Eligible but not included because study is a cross-sectional. |
| Dow et al. (14) | Sugar not reported separately, only as a dietary pattern score. |
| Dhingra et al. (15) | No T2D as an outcome. |
| Drouin-Chartier et al. (16) | Eligible, but SSB was reported as changes in intake over 4 years and not actual intake amounts of SSB. |
| Duan et al. (17) | Eligible, but ORs of T2D for sugary beverages, added sugar and juice in g/d are listed across quintiles of a dietary pattern score. |
| Duffey et al. (18) | No T2D as an outcome. |
| Epic-InterAct Study (19) | Sugar not reported separately, only dietary pattern scores. |
| Ericson et al. (20) | Sugar not reported separately, only as a dietary pattern score. |
| Fantino et al. (21) | Commentary only. |
| Ferreira et al. (22) | T2D incidence not reported. |
| Fresan et al. (23) | Substitution study, measured replacement of water for fresh juice consumption and T2D risk. |
| Hayashino et al. (24) | Only one dose provided for analysis. |
| Hirahatake et al. (25) | Risk ratios for added sugars not reported separately, only combined with saturated fat. |
| Hodge et al. (26) | Only one dose provided for analysis. |
| Horikawa et al. (27) | Sugar only reported by increasing carb intake and not separately. |
| Horikawa et al. (28) | Duplicate of previous Horikawa et al. |
| Huang et al. (29) | Incomplete data: missing cases for SSB. Authors did not respond. |
| Imamura et al. (30) | Quantiles of SSB not reported correctly, only dose response. Study data of 8-country EPIC already included. |
| Jing et al. (31) | Eligible, however this is a cross-sectional study. |
| Jo et al. (32) | T2D incidence not assessed. |
| Khalangot et al. (33) | Quasi-experimental study design. |
| Krishnan et al. (34) | Sugar not assessed in study. |
| La Vecchia et al. | Comment to editor. |
| Lang et al. (35) | Based on surveillance data, sugar, not reported at an individual level. |
| Li et al. (36) | Based on surveillance data, SSB not reported at an individual level. |
| Liu et al. (37) | Sugar not reported in food group categories. |
| Lofvenborg et al. (38) | Case-control study- wrong study design. |
| Lopez et al. (39) | Sugar not reported separately, only as a dietary pattern score. |
| Mandalazi et al. | Sugar not reported separately, only as a dietary pattern score. |
| Mekonnen et al. (40) | Modeling study only. |
| Mohan et al. (41) | Eligible, but this is a cross-sectional study. |
| Muraki et al. (42) | Eligible, but data on cases and participants not provided. Contact with authors was unsuccessful. |
| Muraki et al. (43) | Duplicate of previous Muraki et al. |
| Murray et al. (44) | Eligible, but study reports duplicate data from already included Schulze et al. 2004. |
| Naja et al. (45) | Inappropriate study design: case-control study. |
| Nettleton et al. (46) | Sugar not assessed, only diet soda. |
| Nettleton et al. (47) | Sugar not reported correctly, only as part of dietary pattern. |
| O’Connor et al. (48) | Duplicate data from other O’Connor et al. |
| O’Neill et al. (49) | Abstract only |
| O’Neill et al. (50) | SSB by quantiles of intake not reported. |
| Odegaard et al. (51) | Sugar not reported correctly, only as part of a Western-style diet pattern. |
| Olofsson et al. (52) | Participants had pre-existing T2D |
| Osei et al. (53) | Cross-sectional and dietary pattern analysis. |
| Pan et al. (54) | Study contained duplicate data from already included Bhupathiraju et al |
| Palmer et al. (55) | Duplicate information as Palmer 2008. |
| Pankow et al. (56) | Article not findable, abstract only. |
| Papakonstantinou et al. (57) | Participants had pre-existing diabetes (case-control study design). |
| Parnell et al. (58) | Dietary sugars not assessed. |
| Pereira et al. (59) | Incomplete data. Has risk ratios for fruit juice but number of cases are missing. |
| Perez-Heras et al. (60) | T2D outcome not assessed. |
| Pomares-Millan et al. (61) | T2D incidence not assessed. |
| Qi et al. (62) | Sugar not reported correctly, only as part of dietary pattern score. |
| Ramne et al. (63) | T2D outcome not assessed. |
| Rhee et al. (64) | Sugar not reported correctly, only as part of risk reduction score. |
| Sayhoun et al. (65) | Sugars not reported in study. |
| Shams-White et al. (66) | Sugar not reported correctly, only as part of a risk score. |
| Scheffers et al. (67) | Fruit juice not reported by intake amount, only as a percentage of total juice and SSB intake. |
| Schulze et al. 2004 (68) | Study contained duplicate data from already included Bhupathiraju et al. This study covers years 1991-99 and Bhuhathiraju study covers years 1984-2008. Does not adjust for BMI and energy. |
| Schulze et al. (69) | Repeat of previous Schulze et al. 2004. |
| Schwingshackl et al. (70) | Risk relations of T2D by sugar not reported. |
| Seino et al. (71) | Results for fruit juices not reported. |
| Soto-Estrada et al. (72) | Based on apparent consumption data, sugar not assessed at individual level. |
| Srour et al. (73) | Relative risks of T2D not reported for sugar intake. |
| Sugihiro et al. (74) | Sugar not reported in separate quantiles of intake, only as a single dose. |
| Takeuchi et al. (75) | Participants had pre-existing T2D. |
| Tasevska et al. (76) | Total sugar intake assessed intravenously as biomarker. |
| Teshima et al. (77) | Retrospective cohort design with participants already having impaired glucose tolerance. |
| Tsilas et al. (78) | Systematic review of relevant studies. |
| Van Dam et al. (79) | Dietary sugar assessed as part of a dietary pattern. |
| Van’t Riet et al. (80) | Dietary sugar not assessed. |
| Viana Dias et al. (81) | Eligible, but only two doses of SSBs reported |
| Villegas et al. (82) | Sugar not reported correctly, only as part of dietary pattern score. |
| Vitale et al. (83) | Cross-sectional study and participants have existing T2D |
| Von Ruesten et al. (84) | Eligible, but dose-response data reported for only one dose of SSB and one dose of fruit juice. |
| Voortman et al. (85) | Sugar not assessed separately, only as a part of a dietary guideline adherence score. |
| Wang et al. (86) | Systematic review of relevant studies. |
| Welsh et al. (87) | Duplicate data from Schulze et al. |
| Xi et al. (88) | Systematic review of relevant studies. |
| Yang et al. (89) | Sugar not assessed separately, only as part of a diet score. |
| Yashpal et al. (90) | Sugar not reported correctly, only as part of a dietary pattern score. |
| Zong et al. (91) | No sugar intake reported, only frequency of sugars consumed away from home. |
|  |  |
| **Included studies reviewed and identified as eligible** | **Details on extraction, conversions, and author correspondence** |
| Ahmadi-Abhari et al. (92) | Doses of sugar were reported as percentage of total energy intake and was converted to kilocalories/day. Imaging software was used to extract mean and CIs from graph. Reported intakes were converted and reported statistics were used. |
| Auerbach et al. (93) | Median ounces of juice/d reported. If 23.3g sugar are in one serving (8oz) of fruit juice^1^, we can assume 2.91g sugar per ounce to convert to grams of sugar per day. Reported intakes were converted and reported statistics were used. |
| Bazzano et al. (94) | Median intakes of fruit juices were reported as servings/d. One serving is considered to be 23.3 grams.^1^ Because only quantiles 1 and 5 were provided, the dose-response risk relation by serving of SSB was used in a linear regression model. Reported intakes were converted and reported statistics were used. |
| Bhupathiraju et al. (95) | SSB reported as servings/day. We assumed 1 serving to be 12oz; 12oz = 39 grams of sugar.^2^ Reported intakes were converted and reported statistics were used. |
| Bondonno et al. (96) | Reached out to authors and they responded with the following missing information: OR’s for 5y and 12y quartiles of fruit juice intake, fruit juice intake amount by quartile, cases of T2D by quartile, and number of participants by quartiles for 5y and 12 y follow-up points. 12 year values were used for meta-analysis. Doses given in grams of juice (249 grams of juice converted to 23.3 grams of sugar)^1^. New estimates were used as provided. |
| De Koning et al. (97) | Doses reported as servings of SB per week or month. We assumed 2 servings/month = 24/year = 24/365 servings/day. 2 servings per week is 104/365 servings/day. 6.5/week is (6.5*52)/365 servings/day. One serving of SSB is considered to contain 39 grams of sugar^2^. Reported intakes were converted and reported statistics were used. |
| Eshak et al. (98) | Frequencies of soft drink and fruit juice intake reported for 5-year and 10-year T2D incidence; 10-year values were used only. SSB intakes are reported as rarely, <2 times/wk, 3-4 times/wk and almost every day, which were considered to be 0.25, 1.5, 3.5 and 6 servings per week. Fruit juice was reported as servings per day, converted as 23.3 grams/cup^1^. Reported intakes were converted and reported statistics were used. |
| Fagherazzi et al. (99) | Doses given as mL/wk, however with intervals. For SSB 0, <86, 86-164, 165-359, >359 [mL/wk] à take mean for doses 0, 46,125,257,461. One ml is 0.033814 oz, so for SSB we go into 12 oz servings which are 39g of sugar. Fruit Juice yielded 0, 90, 313.5, 707.5, 1226.6 mL/wk as doses, similarly converted. All values were divided by 7 to obtain intakes per day. Reported intakes were converted and reported statistics were used. |
| Gardener et al. (100) | Detailed frequencies of consumption provided, which we divided into tertiles of soda reported as <1/month, 1/month-6/wk, and daily. Participants were reported as 908, 830, 281 and cases as 154, 160, and 54. From this, doses were 0, 11.13 and 65.23 grams/day (assuming 39 g of sugar per serving)^2^. Reported intakes were converted and reported statistics were used. |
| Janket et al. (101) | Only data for sucrose was used. Authors responded that complete data for other sugars is unavailable. |
| Kanehara et al. (102) | Study provided percent energy intake of sugar/day and mean caloric intake/day from which kcal/day was calculated and divided by 4kcal to find grams of sugar/day. This was done with all reported sugars: total sugar, fructose, sucrose, and glucose. |
| Meyer et al. (103) | First and last quintile doses were given as <31.2 and >51.0. The mean of the first quintile was divided by two, and the last quintile dose response was found by subtracting the 4^th^ quintile mean dose (47.35) from 5^th^ quintile dose (51.0), then adding 5^th^ quintile dose (51.0) to get 54.65 *^4^.* The mean was calculated from the range of doses was given for 2-4 quintiles. |
| Montonen et al. (104) | Relative risks of T2D were reported by quartiles of total sugars, sucrose, fructose, glucose, fruit juice, and SSBs. Fruit juice and SSBs were reported as doses of grams of fluid/day. We used conversions^1,2,3^ to calculate doses as grams of sugar/day. Fruit juice was defined specifically as berry juice. The 4^th^ quartile of total sugar was adjusted by subtracting Q2 from Q3, then adding Q3 to the difference. Our calculations for Q4 resulted in being 157g/day instead of 171 g/day. |
| Mursu et al. (105) | Study was missing for fruit juice intake data so authors were contacted and provided the information (fruit juice intake per quantile). Data was also extracted from published meta-analysis: Xi et al 2014: <https://www.ncbi.nlm.nih.gov/pmc/articles/PMC3969361/> |
| O’Connor et al. (106) | Median intake of grams of fluid (SSB or FJ) were provided. Grams of fluid were converted to grams of sugar^1,2,3^. |
| Odegaard et al. (107) | A range of doses in cups were provided by time. The mean was determined and converted to cups/day. Study stated that one serving of SSB was a value of 237 mL, or one cup (8oz), therefore we modified our conversion 26g of sugar per serving (8 oz). |
| Olsson et al. (108) | Study provided percent energy intake of sucrose and added sugar/day as a range, the mean was determined. Mean caloric intake/day was provided from which kcal/day was calculated and divided by 4kcal to find grams of each sugar/day. Fruit juice and SSBs were provided as a range grams of liquid per day. The mean was found, then calculated to grams of sugar/day^1,2,3^. |
| Palmer et al. (55) | Estimated intakes were based on frequency of servings. Both fruit juice and SSB doses were provided in a range of servings: <1 drink/mo, 1-7 drinks/mo, 2-6 drinks/wk, 1 drink/day, ≥2 drinks/day and were estimated to be 0, 0.115, 0.57, and 1 servings/day. The first 4 quintiles were then translated^1,3^ to grams of sugar, while the 5^th^ quintile was determined by subtracting the 3^rd^ quintile from the 4^th^, and then adding the 4^th^ quintile to the difference.^4^ The same calculation was done for fruit juice. |
| Papier et al. (109) | Study reported doses as rarely, 1-6/wk, and ≥1/day. These were estimated to be 1/mo, 3.5/wk, and 1.5/day. These doses were translated to grams of sugar/day^1,3^. |
| Paynter et al. (110) | Found via manual search. Study provided the same range of doses in cups: <1, 1, 1.1-1.9, and ≥2.0. These were estimated to be 0.1, 1, 1.5, and 2. cups/day respectively. One cup is equivalent to 8 ounces and after converting ounces to grams of sugar^2^, the final doses of sugars in SSB were 2.6, 26, 39, and 52 grams/day. The 4^th^ quartile we calculated by subtracting the 2^nd^ quartile from the 3^rd^ quartile and then adding the 3^rd^ quartile to the difference. For example, 39-26+39 = 52g sugar/day. |
| Ramne et al. (111) | Appears to be the same study as Olsson et al., however this study is based on a subcohort of the MALMO study called the MDC-CC. Doses of sugar were reported as percentage of total energy intake and was converted to kilocalories/day. Kcals/day were converted to grams of sugar/day for both SSBs and added sugar. |
| Rayner et al. (112) | Only data for fruit juice was extracted. Added sugar was removed because it only had two doses which was insufficient for the dose-response meta-analysis. Fruit juice intake was provided in the study as a mean of servings. Total T2D cases were calculated from the percentage of T2D cases multiplied by the total-persons in the given quartile. This study used highest dose as reference for risk ratios, so all risk ratios and confidence intervals were divided by the risk ratio of the lowest dose to make it comparable to other studies. |
| Romaguera et al. (113) | Both fruit juice and SSBs were provided as median doses of grams of liquid/day. These were calculated^1,2,3^ to grams of sugar/day. |
| Sakurai et al. (114) | A range of doses were provided in a range of time. The mean dose and the mean range of time were found, then converted to servings/day. Servings were converted to grams of sugar/day^2^. |
| Scheffers et al. (115) | Grams of liquid/day were provided which were converted to grams of sugar^1^. |
| Schulze et al. (116) | Found via manual search. No conversions needed. |
| Sluijs et al. (117) | No conversions needed. Study is a case-cohort. |
| Stern et al. (118) | Found via manual search. Study provided doses in servings per week. Doses were calculated to grams of sugar/day.^2^ |
| Torres-Ibarra et al. (119) | Doses were converted from servings/wk to grams/day.^2^ |

If studies reported exposure of SSB or fruit juice in serving sizes but did not specify the amount, recommended serving sizes for SSB and fruit juice conversions were used. *1* Conversion of serving into grams based on report from USDA <https://fdc.nal.usda.gov/fdc-app.html#/food-details/169044/nutrients> where 23.3 grams of sugar are in 259 grams of juice (which is one cup, or 8oz).

***2***  One serving of SSB considered to be 12 ounces. 12 ounces considered to contain 39 grams of sugar according to: <https://fdc.nal.usda.gov/fdc-app.html#/food-details/2678649/nutrients> ***3***  12 oz of SSB was reported to be 271g of fluid from: <https://web.physics.ucsb.edu/~lecturedemonstrations/Composer/Pages/36.34.html#:~:text=If%20we%20use%20a%20density,a%20mass%20of%20371%20g> ***4*** For open-ended upper bounds, the difference between the previous group's midpoint and its lower bound was added to the start of the last group to estimate the median (e.g., 15–10=5, added to 20 to yield 25).

*All data were extracted from full-adjusted models containing BMI and other relevant covariates. All conversions were performed by two individual reviewers to check for errors.

# **Supplementary Figures 2 A-B** Risk of publication bias analysis


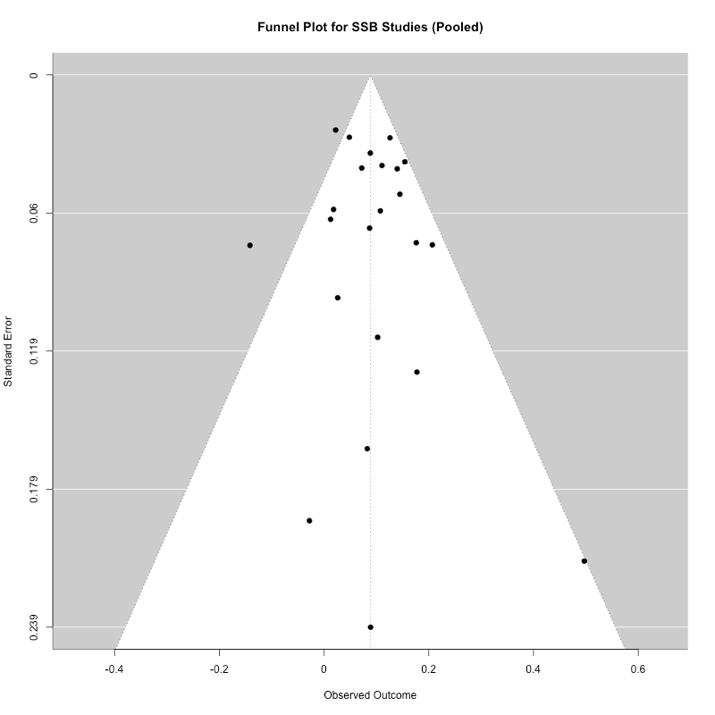


***
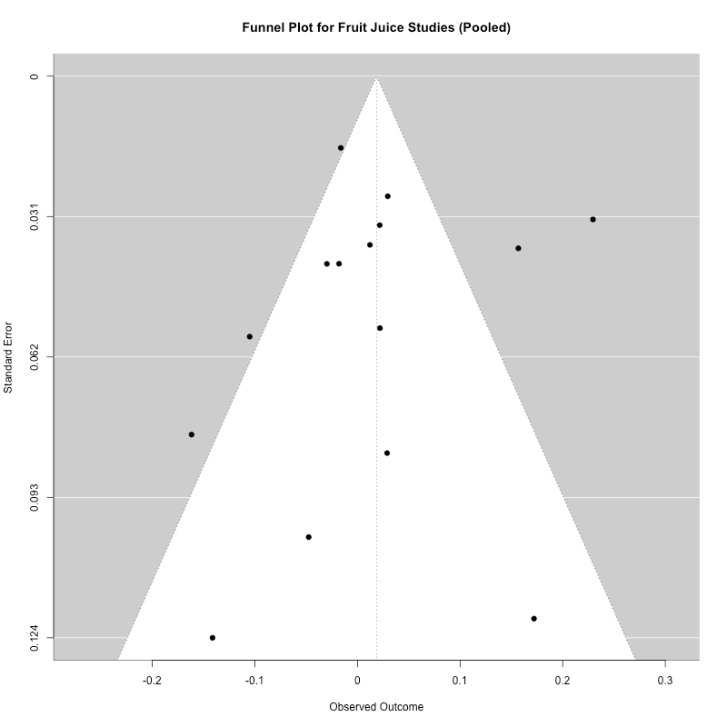
*A B**

Funnel plot for A SSB studies B and fruit juice studies. This funnel plot visualizes potential publication bias across studies examining the relationship between *the exposure sugar category* and T2D Risk Ratios. Each point represents an individual study, plotted by its pooled log risk ratio (x-axis) and standard error (y-axis). Symmetry in the distribution of points suggests the absence of publication bias, while asymmetry may indicate potential bias. This was assessed by visually inspecting for asymmetry, which could indicate selection bias, publication bias, or other influential factors. In Begg's test, the estimated log hazard ratios were plotted against their standard errors (SEs) to determine the correlation (using Kendall’s rank correlation) between the adjusted effect size and the meta-analysis weight. For Egger’s test, the ratio of log hazard ratios to SE was multiplied by 1/SE. This test examined whether the intercept significantly deviated from zero in a regression of standardized hazard ratios against their precision. Publication bias would result in skewness in the plots, and this was considered present if p < 0.10.

# **Supplementary Table 4** Risk of bias judgements for each domain (from the ROBINS-E detailed guidance)

| **Study** | **Bias due to confounding** | **Bias due to exposure assessment** | **Bias due to selection of participants** | **Bias due to misclassification during follow-up** | **Bias due to missing data** | **Bias due to measurement of the outcome** | **Bias due to selective reporting of the results** | **Overall judgement** |
| --- | --- | --- | --- | --- | --- | --- | --- | --- |
| Ahmadi-Abhari et al. (92) | Moderate | Moderate | Moderate | Moderate | Low | Low | Low | **Moderate** |
| Auerbach et al. (93) | M | M | L | M | L | L | L | **Moderate** |
| Bazzano et al. (94) | M | M | L | L | L | L | L | **Moderate** |
| Bhupathiraju et al. (95) | M | M | L | L | L | L | L | **Moderate** |
| Bondonno et al. (96) | M | M | L | M | M | L | L | **Moderate** |
| De Koning et al. (97) | M | M | L | L | L | L | L | **Moderate** |
| Eshak et al. (98) | M | M | L | L | L | L | L | **Moderate** |
| Fagherazzi et al. (99) | M | M | L | M | L | L | L | **Moderate** |
| Gardener et al. (100) | M | M | L | M | L | L | L | **Moderate** |
| Janket et al. (101) | M | M | L | M | L | L | L | **Moderate** |
| Kanehara et al. (102) | M | M | L | L | L | L | L | **Moderate** |
| Meyer et al. (103) | M | M | L | M | L | M | L | **Moderate** |
| Montonen et al. (104) | M | M | M | M | L | L | L | **Moderate** |
| Mursu et al. (105) | M | M | L | M | L | L | L | **Moderate** |
| O’Connor et al. (120) | M | M | L | L | L | L | L | **Moderate** |
| Odegaard et al. (107) | M | M | L | M | L | M | M | **Moderate** |
| Olsson et al. (108) | M | M | L | M | L | L | L | **Moderate** |
| Palmer et al. (55) | M | M | L | L | L | M | L | **Moderate** |
| Papier et al. (109) | M | M | L | L | L | M | L | **Moderate** |
| Paynter (110) | M | M | L | M | M | M | L | **Moderate** |
| Ramne et al. (111) | M | M | L | M | L | L | L | **Moderate** |
| Rayner et al. (112) | M | M | L | L | L | M | L | **Moderate** |
| Romaguera et al. (113) | M | M | L | M | L | L | L | **Moderate** |
| Sakurai et al. (114) | M | M | L | M | L | L | L | **Moderate** |
| Scheffers et al. (115) | M | M | M | M | L | L | L | **Moderate** |
| Schulze et al. (116) | M | M | L | M | L | L | L | **Moderate** |
| Sluijs et al. (117) | M | M | H | M | L | L | L | **High** |
| Stern et al. (118) | M | M | L | M | L | M | L | **Moderate** |
| Torres-Ibarra et al. (119) | M | M | L | L | M | M | L | **Moderate** |

# **Supplementary Table 5** Description and decision criteria for each domain in ROBINS-E

| **Domain** | **Explanation** | **Judgements** |
| --- | --- | --- |
| **Risk of bias due to confounding** | - Is there potential for confounding of the effect of exposure in this study? - Did the authors use a multivariable-adjusted analysis method that controlled for age and markers of overweight/obesity and at least three of the following: sex, family history of diabetes, education/socioeconomic status, smoking, alcohol consumption, physical activity, and total energy intake? - Were confounding factors that were controlled for measured validly and reliably by the variables available in this study? - Did the authors avoid adjusting for post-exposure variables?   *Notes:* **Confounding is expected in all observational studies; thus, no study was assigned low risk of bias**. Time-varying confounding was expected to be unlikely and is not expected to cause risk of bias in the present study. | Low risk of bias:  No bias expected due to confounding, including time-varying confounding.  Some concerns (moderate risk of bias):  Confounding is expected for age, sex, smoking, alcohol consumption, physical activity, weight/body mass index, (and total energy intake), and the authors performed a multivariable-adjusted analysis to control for these confounding factors. The variables adjusted for are valid and reliable measures of the confounding factors.  *Or* Education/socioeconomic status (SES) is not included as confounding factor in the multivariable-adjusted analysis, but SES is not expected to vary substantially within the cohort (e.g. NHS, HPFS).  *Or* The authors statistically investigated whether the confounding factors have an effect on the risk estimate and excluded the confounder from the multivariable model if there was no effect on the overall effect estimate.  High risk of bias:  At least one known important confounding factor was not measured or appropriately controlled for (age and body fat (or BMI or weight).  *Or* The authors adjusted for post-exposure variables that are affected by exposure (e.g. sodium intake and risk of stroke [adjustment for blood pressure during follow-up = intermediate biological variable on the causal pathway] -> overadjustment).  Very high risk of bias:  No adjustment was made at all for any covariate. The authors controlled for post-exposure variables, and the use of negative controls, or other considerations, suggest serious uncontrolled confounding.  No information: No information on which confounders the analysis has been controlled for. |
| **Risk of bias arising from measurement of exposure assessment** | - Does the measured exposure well-characterize the exposure metric specified to be of interest in this study? - Was the exposure likely to be measured with error, or misclassified?   *Notes:* Differential misclassification is not expected to occur in prospective cohort studies, since diet is reported before the occurrence of the outcome (Freedman 2011).  Some type of non-differential misclassification cannot be excluded **(any dietary assessment method involves measurement error), thus no study was assigned low risk of bias.** | Low risk of bias:  Exposure status is well-defined by the measurement and no measurement error is expected in its assessment *or* exposure was measured at multiple times, and exposure is stable or change only slightly over time *or* exposure was measured with a single measurement, but exposure can be assumed to be stable over time.  Some concerns (moderate risk of bias):  Exposure status is well-defined by the measurement, and exposure was measured using an established or validated tool (e.g., a validated FFQ, multiple 24h recalls) *and* exposure was measured at multiple times, and exposure is stable or change only slightly over time *or* exposure was measured at one single point of time, but exposure can be assumed to be stable over time.  High risk of bias:  Exposure status is not well characterized by the measurement (e.g., assumed from an indirect measurement)  *and/or* exposure was measured using a not validated tool  *and/or* exposure was measured with a single measurement, which is unlikely to characterize exposure during the period (e.g., single 24h recall) or exposure cannot be assumed to be stable over time.  No information: No definition of exposure or no explanation of the source of information about exposure status is reported. |
| **Risk of bias in selection of participants into the study** | - Was selection of participants into the study (or into the analysis) based on participant characteristics observed after the start of the exposure window being studied? - Do start of follow-up and start of exposure coincide for most participants?   Notes: In observational studies, it is unlikely that post-exposure variables influenced selection of participants into the study. Exclusion of participants may be mostly based on missing data, which will be considered in the domain referring to missings (see below). The start of follow-up is considered to coincide with the baseline exposure assessment. However, participants are already exposed at start of the study, which might have influenced outcome measured that occurred shortly after start of the study. | Low risk of bias:  All participants who would have been eligible for the target study were included in the study *and* start of exposure and follow-up coincide.  Some concerns (moderate risk of bias):  Selection into the study may have been related to exposure and outcome (e.g. inclusion of postmenopausal women only); *and* the authors used appropriate methods to correct for the selection bias *and/or* start of exposure and follow-up do not coincide, but the association of exposure is constant over time.  High risk of bias:  Selection into the study was related to exposure and outcome *and/or* start (e.g. only participants with prediabetes were included in the analysis for type 2 diabetes, or specific patients groups for analysis on mortality) and *and* this could not be corrected for in the analyses;  *or* exposure and follow-up do not coincide and the effect of exposure (rate ratio) is not constant over time.  No information: No information is reported about selection of participants into the study. |
| **Bias due to misclassification during follow-up or post-exposure interventions** | - Were there deviations from the exposure beyond what would be expected in usual practice? - Were these deviations unbalanced among participants and likely to have affected the outcome?   Notes: Repeated measurements of the exposure are mostly not available in observational studies. It is not expected that there are high changes in diet in healthy participants. Changes in diet may be similar between studies and may also be similar between groups (differential misclassification is not expected). Recent studies have shown that diet is constant or change only slightly over time (Feldman 2017 IJBNPA, VanWormer 2017 Preventive Medicine Reports). Thus, if repeated measures are not available, moderate risk of bias could be assigned to a study. | Low risk of bias:  Repeated measurements of the exposure status during follow-up are available. No or only slight changes were observed and the changes were considered in the analysis.  Some concerns (moderate risk of bias):  Repeated measurements of the exposure are not available, but high changes are not expected during follow-up (compare notes)  or repeated measurements of the exposure status during follow-up are available and some changes in lifestyle factors were observed. The analysis was appropriate to estimate the effect of changes in lifestyle factors, allowing for deviations that were likely to impact the outcome;  High risk of bias:  Exposure status is measured during follow-up and high changes in lifestyle factors have been observed, and the analysis was not appropriate to estimate the effect of changes in lifestyle factors, allowing for deviations that were likely to impact on the outcome.  No information: No information on deviations from the exposure is reported. |
| **Bias due to missing data** | - Were there missing outcome data? - Were participants excluded due to missing data on exposure status? - Were participants excluded due to missing data on other variables needed for analysis?   *Notes:* Missing data on exposure variables and other variables are expected to be missing at random and not related to exposure or outcome that have been assessed during follow-up. | Low risk of bias:  Little loss-to-follow-up (<20%) and data on exposure and other variables were reasonably complete (<10% missing data) and was unlikely to introduce bias.  *or* the analysis addressed missing data and is likely to have removed any risk of bias.  Some concerns (moderate risk of bias):  There is a proportion of missing data in the original cohort or a high proportion of loss-to-follow-up *and* the analysis is unlikely to have removed the risk of bias arising from the missing data (e.g., using logistic regression).  High risk of bias:  High proportions (>50%) of missing data *and* the analysis is unlikely to have removed the risk of bias arising from the missing data  *or* missing data were addressed inappropriately in the analysis;  *or* the nature of the missing data means that the risk of bias cannot be removed through appropriate analysis.  No information: No information is reported about missing data or the potential for data to be missing. |
| **Risk of bias due to measurement of the outcome** | - Were the methods of outcome assessment comparable across exposure groups? - Could the outcome measure have been influenced by knowledge of the exposure status? - Were any systematic error in measurement of the outcome related to exposure status?   *Notes:* In observational studies, it is not expected that outcome assessors were aware of exposure status of the participants. | Low risk of bias:  The methods of outcome assessment were comparable across exposure groups *and* the outcome measure was unlikely to be influenced by knowledge of the exposure status of study participants *and* any error in measuring the outcome is unrelated to exposure status (i.e. objective measures or self-reported outcomes that are (mostly, ≥90%) confirmed by a second source ,e.g., medical records, record linkage and death certificates).  Some concerns (moderate risk of bias):  The methods of outcome assessment were comparable across exposure groups *and* any error in measuring the outcome may be minimally related to exposure status *or* outcome measure are not reliable measured (i.e. confirmed records are available for <90% of all participants and the authors did not perform an additional analysis separating confirmed and probable cases).  High risk of bias:  The methods of outcome assessment were not comparable across exposure groups *or* the outcome measure was subjective (i.e. self-report of CVD, type 2 diabetes, etc. by study participants or next of kin, without confirmation by a second source) *and* error in measuring the outcome was related to exposure status.  No information: No information is reported about the methods of outcome assessment. |
| **Risk of bias due to selection of the reported result** | - Was the result reported in accordance with an available, pre-determined analysis plan? - Is the reported effect estimate likely to be selected from multiple analyses of exposure-outcome relationship? - Is the reported effect estimate likely to be selected from different subgroups?   *Notes:* In observational studies, it is unusual to publish an a priori analysis plan or protocol. Multiple outcome measurements for the definition of CVD, mortality, type 2 diabetes, etc. are not expected. | Low risk of bias:  There is a clear description of all analysis and the analyses are consistent, and all reported results correspond to all intended outcomes, analyses and sub-cohorts.  Some concerns (moderate risk of bias):  The results are reported according to an a-priori analysis plan or protocol, and there is indication of selection of the reported analysis among multiple analyses; *or* there is indication of selection of the cohort or subgroups for analysis and reporting on basis of the results (e.g. estimates not shown for all analyses).  High risk of bias:  There is a high risk of selective reporting from multiple exposure measurements, *or* outcomes measurements, *or* multiple analyses of data *or* the cohort or subgroup is selected from a larger study for analysis and appears to be reported based on the results.  No information: There is too little information to make a judgement. |
| **Overall judgement** | Low risk of bias | The study is judged to be at low risk of bias for all domains. |
|  | Some concerns (moderate risk of bias) | The study is judged to be at low risk of bias or some concerns (moderate) for all domains. |
|  | High risk of bias | The study is judged to be at high risk of bias in at least one domain, but no domains are at very high risk of bias. |
|  | Very high risk of bias | The study is judged to be at very high risk of bias in at least one domain. |

| **Supplementary Table 6** Study characteristics | | | | | | | | | | | | |
| --- | --- | --- | --- | --- | --- | --- | --- | --- | --- | --- | --- | --- |
| **First author (year)** | **Country** | **Date source** | **Mean/median age and age range** | **% Men** | **Total number** | **Total cases** | **Median Follow up Years** | **Exposure** | **Exposure measure** | **HR/RR/OR** | **Outcome assessment** | **Adjustment** |
| Ahmadi-Abhari (2014) (92) | England | EPIC-Norfolk | 40-79y | 56.9 | 25,639 | 749 | 6.2 | Sucrose  Glucose  Fructose  TS | Validated SFFQ, 7-d food diary | HR | Medical record linkage | Age, sex, total energy intake, BMI, family history of diabetes, cigarette smoking, alcohol intake, physical activity, education level. |
| Auerbach  (2017) (93) | USA | Women’s Health Initiative (WHI) | 50-79y | 0 | 114,219 | 11,488 | 7.8 | FJ | Validated SFFQ | HR | Self-report validated by medical records, diabetes medicine use | Age, education level, race/ethnicity, smoking, physical activity, BMI, hormone replacement therapy, study arm, and energy intake. |
| Bazzano (2008) (94) | USA | Nurses’ Health Study I (NHSI 1984-2002) | 40-69y | 0 | 71,346 | 4529 | 14 | FJ | Validated SFFQ | HR | Self-report, validated by medical records | Age, BMI, physical activity, family history of diabetes, postmenopausal hormone use, alcohol use, smoking, and total energy intake, whole grains, nuts, processed meats, coffee, potatoes, SSB. |
| Bhupathiraju (2013)^a^ (95) | USA | Nurses Health Study (NHS 1984-2008) | 30-55y | 0 | 74,749 | 7370 | 24 | SSB | Validated FFQ | RR | Self-report validated by medical records | Age, time interval, smoking status, alcohol use, postmenopausal hormone use, physical activity, family history of diabetes, alternate Healthy Eating Index, consumption of other beverages other than the main exposure, hypertension, hypercholesterolemia, adherence to a low-calorie diet, reported weight change, weight gain, weight loss, total energy intake, BMI. |
| Bhupathiraju (2013)^a^ (95) | USA | Health Professional Follow-up Study (HPFS 1986-2008) | 40-75y | 100 | 39,059 | 2865 | 22 | SSB | Validated FFQ | RR | Self-report validated by medical records | Age, time interval, smoking status, alcohol use, postmenopausal hormone use, physical activity, family history of diabetes, alternate Healthy Eating Index, consumption of other beverages other than the main exposure, hypertension, hypercholesterolemia, adherence to a low-calorie diet, reported weight change, weight gain, weight loss, total energy intake, and BMI. |
| Bondonno  (2021) (96) | Australia | Australian Diabetes, Obesity and Lifestyle Study (ADOLS) | 54y | 45 | 3,518 | 247 | 12 | FJ | SFFQ (validated previously by Hodge et al.) | OR | Biomarkers used ( ≥7.0 mmol/L, plasma glucose of ≥ 11.1 mmol/L) or medicine use) | Age, sex, physical activity levels, education level, socio-economical index, income, BMI, smoking status, CVD, family history of diabetes, energy intake, and intakes of alcohol, vegetables, red meat, and processed meat. |
| De Koning  (2011) (97) | USA | Health Professionals Follow-Up Study (HPFS) | 40-75y | 100 | 40,389 | 2680 | 20 | SSB | Validated SFFQ | HR | Self-report and validated with medical records | Age, smoking, physical activity, alcohol intake multivitamin use, family history, high triglycerides at baseline, high blood pressure, diuretics, pre-enrollment weight change, dieting, total energy, BMI. |
| Eshak  (2013) (98) | Japan | Japan Public Health Center-based Prospective Study (JPHC) | 40-59y | 43.9 | Men: 12,137  Women:  15,448 | Men: 484  Women: 340 | 10 | FJ  SSB | Validated FFQ | OR | Self-report validated by medical records | Age, BMI, family history of diabetes mellitus, education, occupation, smoking status, alcohol intake, history of hypertension, leisure-time physical activity, consumption of coffee, consumption of green tea, energy-adjusted intakes of dietary magnesium, calcium, vitamin D, rice and total dietary fiber, and total energy intake. |
| Fagherazzi (2013) (99) | Europe | Etude Epidémiologique auprès des femmes de la Mutuelle Générale de l'Education Nationale (E3N) | 52.8y | 0 | 66,118 | 1369 | 14 | FJ  SSB | Validated 208-item diet-history questionnaire | HR | Self-report, validated by records | (Age), education, smoking status, physical activity, hypertension, hypercholesterolemia, use of hormone replacements, family history of diabetes, antidiabetic drugs, intakes of alcohol, omega-3, carbohydrate, coffee, fruit and vegetables, processed-meat; dietary pattern, total energy intake and BMI. |
| Gardener  (2018) (100) | USA | Northern Manhattan Study (NOMAS) | 69y | 36 | 2019 | 368 | 11 | SSB | Validated FFQ | HR | Self-report and validated by medical records | Age, sex, race/ethnicity, Mediterranean diet, total calories, smoking, physical activity, alcohol use, BMI, hypertension, hypercholesterolemia, and calorie (energy) consumption. |
| Janket (2003) (101) | USA | WHS | 46-61y | 0 | 38,480 | 918 | 6 | Sucrose | Validated SFFQ | RR | Self-report validated by medical records | Age, smoking, BMI, vigorous exercise, alcohol use, history of hypertension, history of high cholesterol, postmenopausal hormone use, vitamin use, and family history of type 2 diabetes. All nutrients were energy adjusted. |
| Kanehara  (2021) (102) | Japan | Japan Public Health Center-based Prospective Study (JPHC) | 40-69y | 43 | 64,677 | 1190 | 10 | Sucrose  Glucose  Fructose  TS | Validated FFQ | OR | Self-report of physician- diagnosed were validated in sub-cohort by medical records | Age, public health center area, occupation, family history of diabetes, history of hypertension, smoking status, alcohol consumption, physical activity, total energy intake, calcium, magnesium, vitamin D, dietary fiber, and coffee consumption, number of births (for women), and starch intake, BMI. |
| Meyer (2000) (103) | USA | IWHS | 55-69y | 0 | 35,988 | 1141 | 6 | Sucrose  Glucose  Fructose | Validated FFQ | RR | Self-report of physician- diagnosed cases validated in subcohort | Age, total energy intake, BMI (quintiles), waist-to-hip ratio (quintiles), education, pack-years of smoking, alcohol intake, and physical activity. |
| Montonen (2007) (104) | Finland | FMHCES | 40-69y | 53.1 | 4284 | 177 | 12 | Sucrose  Glucose  Fructose  TS  Berry Juice  SSB | Diet history (Interview with questionnaire) | RR | Self-report and validated by medical records | Age, sex, BMI, energy intake, smoking status, geographical area, physical activity, family history of diabetes, prudent dietary pattern score, conservative pattern score, serum cholesterol, blood pressure, history of infarction, history of angina pectoris, and history of cardiac failure. |
| Mursu (2014) (105) | Finland | Kuopio Ischaemic Heart Disease Risk Factor Study (KIHD) | 42-60y | 100 | 2332 | 432 | 19.3 | FJ | 4-day diet record | HR | Self-report verified by medical records | Age, examination years, BMI, waist-to-hip ratio, smoking, education, physical activity, family history of diabetes, intakes of energy and alcohol. |
| O’Connor (120) | England | EPIC-Norfolk study | 40-79y | 45.3 | 25,639 | 847 | 10.8 | SSB  FJ | 7-day food diary | HR | Self-report verified by medical record linkage | Age, sex, social class, education level, family history of diabetes, physical activity level, smoking status, alcohol intake, other sweet beverages, season, BMI, total energy intake. |
| Odegaard (2010) (107) | Singapore | Singapore Chinese Health Study (SCHS) | 54.8y | 42.9 | 43,580 | 2273 | 5.7 | FJ  SSB | Validated FFQ | RR | Self-report, validated by use of oral/insulin medications | Age, sex, dialect, year of interview, educational level, smoking status, alcohol use, physical activity, intakes of saturated fat, fiber, dairy, juice or soft drink intake depending on model, and coffee; BMI and energy intake. |
| Olsson  (2021) (108) | Sweden | Malmö Diet and Cancer Study (MDC) | 57.75 | 39 | 26,622 | 4046 | 18 | Sucrose  FJ  SSB  Added sugar | 7-d food diary and Validated FFQ | HR | Medical record linkage | Age, sex, diet method version, season, total energy intake, physical activity, alcohol habits, smoking, education, coffee, meat, whole grains and SSB depending on model and BMI. |
| Palmer (2008) (55) | USA | Black Women’s Health Study (BWHS) | 21-69y | 0 | 43,960 | 2713 | 10 | FJ  SSB | Validated FFQ | HR | Self-report and validated by medical records | Age, family history of diabetes, physical activity, cigarette smoking, years of education, other drink types, intake of red meat, processed meats, cereal fiber, and coffee, and glycemic index. |
| Papier  (2017) (109) | Thailand | Thai Cohort Study  (TCS) | University students | 45 | 39,175 | 695 | 8 | SSB | Questionnaire on SSB frequency validated in study (Lin et al. Plos One, 2014) | OR | Self-report validated by medical records | Age, BMI, hypertension, residence, education, income, physical activity, smoking, alcohol, intake of fruit, vegetables, and deep-fried food. |
| Paynter  (2006)(110) | USA | Atherosclerosis Risk in Communities Study (ARIC) | 45-64y | 44 | 12,204 | 1437 | 9 | SSB | SFFQ (validated previously by Willet et al.) | HR | Biomarkers, use of T2D medications | Age, study center, race, education, and family history of diabetes, BMI, waist:hip ratio, total caloric (energy) intake, dietary fiber, smoking, alcohol consumption, leisure activity, and hypertension. |
| Ramne  (2020) (111) | Sweden | Malmö Diet and Cancer–Cardiovascular Cohort  (MDC-CC) | 57y | 40 | 4382 | 750 | NI | Added sugar  SSB | Validated 7-d food record, FFQ, and interview | HR | Medical record linkage | Age, sex, diet method version, season, total energy intake, physical activity, alcohol habits, smoking, education and BMI. |
| Rayner (2020) (112) | Australia | Australian Longitudinal Study on Women’s Health (ALSWH) | 53y | 0 | 9689 | 959 | 15 | FJ | Validated FFQ | RR | Self-report validated by medical records | Age, country of birth, total energy intake, highest educational qualification, employment status, years of follow-up, history of gestational diabetes mellitus (GDM), physical activity, and BMI. |
| Romaguera (2013) (113) | Europe | European Prospective Investigation into Cancer and Nutrition Study (EPIC) -InterAct | 52.4y | 37.8 | 15,374 | 11,684 | 10-16 | FJ  SSB | Country-specific validated dietary questionnaire | HR | Medical records, medication use | Age (as underlying timescale), sex, educational level, physical activity, smoking status, energy intake, BMI and alcohol consumption; juices and total soft drinks. |
| Sakurai (2014) (114) | Japan | Japan Occupational Cohort (JOC) | 35-55y | 100 | 2,037 | 170 | 5.5 | SSB | Validated diet history questionnaire | HR | Biomarkers and T2D medication use | Age, BMI, family history of diabetes, smoking, alcohol, exercise, hypertension, dyslipidemia, on a diet for chronic disease, and intakes of total energy, fiber, diet soft drinks, fruit juice, vegetable juice, and coffee. |
| Scheffers (2020) (115) | Nether-lands | EPIC – Netherlands (EPIC-NL) | 20-69y | 68 | 36,147 | 1477 | 14.6 | FJ | Validated FFQ | HR | Self-report verified by medical records | Age, sex, educational level, physical activity, smoking, family history of diabetes, DHD15-index, alcohol, coffee, sugar-sweetened beverages, fruit, BMI, and waist circumference. |
| Schulze (2008) (116) | Germany | EPIC – Potsdam | 35-65y | 38.7 | 27,548 | 844 | 7-11 | Sucrose  Glucose  Fructose | Validated SFFQ | RR | Self-report confirmed validated by medical records | Age, sex, education, occupational activity, sport activity, cycling, smoking, alcohol intake, energy intake, BMI, waist circumference, fiber intake, Mg intake, PUFA:SFA ratio, and MUFA:SFA ratio. |
| Sluijs  (2013)(117) | Europe | EPIC – Interact Study | 52y | M + F | 15,258 | 11,559 | 12 | TS | Validated dietary questionnaire or SFFQ depending on country | HR | Medical record linkage and medication use | Age (as underlying timescale), center, sex, education, physical activity, BMI, menopausal status, smoking status, alcohol consumption, dietary intake of total energy, protein, polyunsaturated: saturated fat ratio, and fiber. |
| Stern  (2019) (118) | Mexico | MTC (Mexican Teachers’ Cohort) | ≥25y | 0 | 72,667 | 3155 | 2.16 | SSB | Validated SFFQ | HR | Self-report validated by medication use | Age, region, SES, family history of diabetes, smoking status, physical activity, food groups: fruits, vegetables, red meat, processed meat, whole grains, and juice. |
| Torres-Ibarra (2020) (119) | Mexico | Health Workers Cohort Study | 44 ± 12.5y | 24.4 | 1445 | 109 | 6.7 | SSB | Validated SFFQ | HR | Biomarkers, physician diagnosis, or medication use | Age, sex, education level, total energy intake, smoking status, physical activity, family history of diabetes, alcohol, hypertension status, BMI. |

# **Supplementary Table 7 A-G** Outcome statistical data and intake amounts by sugar category

|  |  |  | |  | |  | |  | |  |  | |  | | | |  |  |  |  |
| --- | --- | --- | --- | --- | --- | --- | --- | --- | --- | --- | --- | --- | --- | --- | --- | --- | --- | --- | --- | --- |
| First author  (condition) | Study period | | Quantile | | Exposure (g/day) | |  | | Ratio |  | | Cases | | Non-cases or Person years* | | | | | |  |
| A Studies used in the primary analysis of **sucrose** | | | | | | | | | | | | | | | | |  |  |  |  |
|  |  | |  | |  | | *Median* | | L95%CI | U95%CI | |  | |  | |  |  |  |  |  |
| Ahmadi-Abhari | 1993-2006 | | 1  2  3  4  5 | | 24.86  36.73  45.00  53.80  69.28 | | 1.0  0.87  0.84  0.98  0.91 | | 0.65  0.61  0.72  0.67 | 1.15  1.12  1.31  1.22 | | 184  147  124  144  154 | | | 662^†^  677  669  702  690 | | | | |  |
| Janket | 1993-2000 | | 1  2  3  4  5 | | 25.8  33.6  39.3  45.8  57.2 | | 1.0  1.0  0.98  1.0  0.84 | | 0.81  0.79  0.81  0.67 | 1.23  1.22  1.24  1.04 | | 196  194  175  188  165 | | | 44,362*  44,298  44,549  44,567  44,476 | | | | |  |
| Kanehara (Men) | 1990-2000, 1993-2003 | | 1  2  3  4 | | 10.30  19.46  29.07  46.93 | | 1.0  1.03  0.81  0.97 | | 0.83  0.63  0.73 | 1.29  1.04  1.28 | | 197  190  142  161 | | | 6752^†^  6759  6808  6788 | | | | |  |
| Kanehara (Women) | 1990-2000, 1993-2003 | | 1  2  3  4 | | 12.72  21.31  30.08  45.00 | | 1.0  0.97  1.01  1.17 | | 0.75  0.76  0.87 | 1.26  1.34  1.57 | | 137  118  116  129 | | | 9083^†^  9102  9104  9091 | | | | |  |
| Meyer | 1986-1992 | | 1  2  3  4  5 | | 15.60  34.60  40.85  47.35  54.65 | | 1.00  0.98  0.96  0.93  0.81 | | 0.82  0.79  0.76  0.67 | 1.19  1.16  1.13  0.99 | | 245  236  230  220  210 | | | 40,082*  40,650  40,824  40,710  40,387 | | | | |  |
| Montonen | 1967-1979 | | 1  2  3  4 | | 28.5  43.2  56.7  79.5 | | 1.0  1.21  1.33  1.12 | | 0.79  0.88  0.71 | 1.87  2.02  1.76 | | 42  43  51  39 | | | 1023^†^  1028  1023  1035 | | | | |  |
| Olsson | 1991-2016 | | 1  2  3  4  5 | | 17.95  37.61  46.72  57.27  141.60 | | 1.0  0.91  1.06  0.96  1.00 | | 0.83  0.96  0.87  0.91 | 1.01  1.17  1.06  1.11 | | 897  762  838  755  794 | | | 95,965*  99,788  98,596  99,014  95,823 | | | | |  |
| Schulze  (Men) | 1994-2005 | | 1  2  3  4  5 | | 22.5  37.6  51.0  67.7  102.0 | | 1.0  0.96  0.98  0.85  0.72 | | 0.73  0.74  0.62  0.50 | 1.26  1.31  1.17  1.04 | | 119  109  101  83  79 | | | 13,715*  13,541  13,555  13,577  13,397 | | | | |  |
| Schulze  (Women) | 1994-2005 | | 1  2  3  4  5 | | 28.2  39.3  48.5  60.3  83.4 | | 1.0  0.89  0.86  0.90  1.13 | | 0.64  0.60  0.62  0.74 | 1.24  1.22  1.31  1.74 | | 85  69  62  61  78 | | | 21,871*  21,740  21,963  21,609  21,497 | | | | |  |

|  | | | | | | | | | | | | | | |
| --- | --- | --- | --- | --- | --- | --- | --- | --- | --- | --- | --- | --- | --- | --- |
| First author | |  | | Quantile | Exposure (g/day) | | |  | | Ratio |  | Cases | Non-cases or Person years* | |
| B Studies used in the primary analysis of **fructose** | | | | | | | | | | | | | | |
|  |  | |  | | |  | *Median* | | L95%CI | | U95%CI |  | | |
| Meyer | | 1986-1992 | | 1  2  3  4  5 | 7.95  18.1  22.45  27.3  32.7 | | | 1.00  0.95  1.17  1.18  1.27 | | 0.77  0.96  0.97  1.06 | 1.16  1.42  1.43  1.54 | 216  200  230  232  263 | 39,897*  40,929  40,865  40,641  40,322 | |
| Ahmadi-Abhari | | 1993-2006 | | 1  2  3  4  5 | 7.95  13.22  17.42  21.71  28.98 | | | 1.0  0.75  0.68  0.76  0.65 | | 0.58  0.52  0.58  0.47 | 0.97  0.89  1.00  0.88 | 207  147  138  146  115 | 673^†^  683  688  685  671 |  |
| Kanehara (Men) | | 1990-2000, 1993-2003 | | 1  2  3  4 | 10.30  18.32  26.11  39.62 | | | 1.0  0.98  0.87  0.99 | | 0.78  0.68  0.76 | 1.22  1.12  1.3 | 196  177  152  165 | 6753^†^  6772  6798  6784 |  |
| Kanehara (Women) | | 1990-2000, 1993-2003 | | 1  2  3  4 | 12.72  20.82  29.04  42.23 | | | 1.0  1.06  0.93  1.29 | | 0.82  0.7  0.95 | 1.38  1.25  1.73 | 132  126  102  140 | 9088^†^  9094  9188  9080 |  |
| Montonen | | 1967-1979 | | 1  2  3  4 | 6.0  11.3  17.0  28.8 | | | 1.0  1.08  1.11  1.52 | | 0.69  0.71  1.00 | 1.67  1.75  2.32 | 40  41  39  55 | 1033^†^  1029  1029  1018 |  |
| Schulze  (Men) | | 1994-2005 | | 1  2  3  4  5 | 8.4  14.4  19.9  26.6  40.6 | | | 1.0  1.03  0.84  0.94  1.00 | | 0.78  0.63  0.70  0.74 | 1.36  1.14  1.27  1.35 | 111  97  82  89  112 | 13,715*  13,541  13,555  13,577  13,397 |  |
| Schulze  (Women) | | 1994-2005 | | 1  2  3  4  5 | 11.0  15.8  19.9  25.0  34.8 | | | 1.0  1.18  1.04  0.76  1.09 | | 0.85  0.73  0.52  0.75 | 1.65  1.46  1.11  1.58 | 78  72  71  55  79 | 21,871*  21,740  21,963  21,609  21,497 |  |

|  |  |  |  |  |  |  |  |  |
| --- | --- | --- | --- | --- | --- | --- | --- | --- |
| First author |  | Quantile | Exposure (g/day) |  | Ratio |  | Cases | Non-cases or Person years* |
| C Studies used in the primary analysis of **total sugars** | | | | | | | | |
|  |  |  |  | *Median* | L95%CI | U95%CI |  |  |
| Ahmadi-Abhari | 1993-2006 | 1  2  3  4  5 | 71.09  93.54  106.46  118.91  135.40 | 1.0  0.94  0.80  0.85  0.85 | 0.71  0.60  0.63  0.63 | 1.24  1.07  1.15  1.15 | 191  160  132  139  131 | 660^†^  687  682  684  687 |
| Kanehara (Men) | 1990-2000, 1993-2003 | 1  2  3  4 | 32.53  50.95  67.64  95.08 | 1.0  0.93  0.91  0.91 | 0.74  0.71  0.68 | 1.16  1.17  1.21 | 198  173  162  157 | 6751^†^  6776  6788  6784 |
| Kanehara (Women) | 1990-2000, 1993-2003 | 1  2  3  4 | 35.53  54.23  71.57  98.34 | 1.0  1.08  1.02  1.24 | 0.83  0.76  0.91 | 1.41  1.37  1.70 | 139  126  110  125 | 9081^†^  9094  9110  9095 |
| Montonen | 1967 - 1979 | 1  2  3  4 | 92.0  115  136  171 | 1.0  1.32  1.07  1.44 | 0.87  0.68  0.93 | 2.01  1.69  2.23 | 43  47  37  48 | 1023^†^  1021  1038  1027 |
| Sluijs | 1991-2007 | 1  2  3  4 | 65  88  108  137 | 1.0  0.95  0.86  0.90 | 0.84  0.78  0.80 | 1.08  0.94  1.03 | 3251  2872  2741  2695 | 564^†^  942  1074  1119 |

|  |  |  |  |  |  |  |  |  |
| --- | --- | --- | --- | --- | --- | --- | --- | --- |
| First author |  | Quantile | Exposure (g/day) |  | Ratio |  | Cases | Non-cases or Person years* |
| D Studies used in the primary analysis of **added sugars** | | | | | | | | |
|  |  |  |  | *Median* | L95%CI | U95%CI |  |  |
| Olsson | 1991-2016 | 1  2  3  4  5 | 18.80  43.31  54.70  68.09  197.72 | 1.0  0.94  0.97  0.97  0.95 | 0.85  0.88  0.88  0.85 | 1.03  1.06  1.07  1.04 | 892  795  806  785  768 | 97,055*  98,508  99,560  98,001  96,061 |
| Ramne | 1991-2016 | 1  2  3  4  5  6 | 13.09  34.77  50.27  72.92  109.68  144.68 | 1.0  0.81  0.80  0.81  0.83  1.01 | 0.62  0.61  0.62  0.59  0.63 | 1.05  1.01  1.02  1.13  1.64 | 80  137  186  260  66  20 | 312^†^  705  943  1284  316  73 |

|  |  |  | |  |  | |  | |  | | |  | | |  |  |
| --- | --- | --- | --- | --- | --- | --- | --- | --- | --- | --- | --- | --- | --- | --- | --- | --- |
| First author | Study period | | Quantile | Exposure (g/day) | |  | | Ratio |  | | Cases | | | Non-cases or Person years* | |  |
| E Studies used in the primary analysis of **fruit juice** | | | | | | | | | | | | | | | |  |
|  |  | |  |  | | *Median* | | L95%CI | U95%CI |  | | | |  | |  |
| Auerbach | 1993-2005 | | 1  2  3  4  5 | 0  1.91  7.57  14.26  22.70 | | 1.0  0.98  0.99  1.00  0.97 | | 0.92  0.93  0.93  0.91 | 1.04  1.05  1.07  1.03 | 1435  2529  2522  2541  2461 | | | 102,874*  183,543  183,980  183,210  184,126 | | |  |
| Bazzano | 1984-2002 | | 1  2  3  4  5 | 0.93  7.32  14.84  22.35  30.99 | | 1.0  1.20  1.28  1.25  1.33 | | 1.09  1.16  1.13  1.20 | 1.32  1.40  1.38  1.48 | 749  946  1,032  920  882 | | | 13,824^†^  13,462  13,305  13,198  13,028 | | |  |
| Bondonno | 1999-2012 | | 1  2  3  4 | 0.25  1.60  7.29  19.74 | | 1.0  1.19  1.21  1.17 | | 0.87  0.86  0.80 | 1.61  1.70  1.70 | 64  59  65  59 | | | 827^†^  820  868  756 | | |  |
| Eshak  (Men) | 1990-2000 | | 1  2  3  4 | 0.83  4.98  11.62  19.92 | | 1.0  0.81  0.93  1.17 | | 0.65  0.65  0.69 | 1.01  1.35  2.00 | 302  129  36  17 | | | 6813^†^  3615  878  347 | | |  |
| Eshak  (Women) | 1990-2000 | | 1  2  3  4 | 0.83  4.98  11.62  19.92 | | 1.0  0.94  0.90  1.37 | | 0.73  0.58  0.79 | 1.21  1.40  2.37 | 198  99  25  18 | | | 8877^†^  4517  1173  541 | | |  |
| Fegharazzi | 1993-2007 | | 1  2  3  4  5 | 0  1.27  4.41  9.95  17.26 | | 1.0  0.90  0.95  1.18  0.93 | | 0.76  0.81  1.01  0.78 | 1.07  1.12  1.38  1.10 | 522  200  199  246  202 | | | 299,619*  136,050  135,111  134,251  133,066 | | |  |
| Montonen | 1967-1979 | | 1  2  3  4 | 0  0.71  1.97  4.77 | | 1.0  0.66  0.93  1.53 | | 0.40  0.59  1.07 | 1.10  1.46  2.18 | 70  19  27  59 | | | | 1595^†^  707  775  1032 | |  |
| Mursu | 1984-2009 | | 1  2  3  4 | 0  3.68  11.93  35.93 | | 1.0  1.07  1.03  0.99 | | 0.82  0.78  0.74 | 1.39  1.34  1.31 | 222  75  71  64 | | | 1002^†^  294  295  309 | | |  |
| O’Connor | 1993-2006 | | 1  2  3  4 | 0  1.97  7.21  16.38 | | 1.0  0.81  0.94  0.99 | | 0.65  0.76  0.80 | 1.01  1.16  1.22 | 524  97  109  117 | | | | 12,930^†^  3752  3509  3615 | |  |
| Odegaard | 1993-2004 | | 1  2  3  4 | 0  1.66  3.32  12.61 | | 1.0  1.0  0.94  1.24 | | 0.87  0.75  1.01 | 1.16  1.18  1.53 | 1871  223  80  99 | | | 205,272*  24,603  10,030  9,269 | | |  |
| Olsson | 1991-2016 | | 1  2  3  4 | 0  1.91  8.55  18.19 | | 1.0  1.04  1.02  1.03 | | 0.95  0.93  0.94 | 1.13  1.11  1.12 | 1852  755  703  736 | | | 209,207*  93,511  90,499  95,968 | | |  |
| Palmer | 1995-2005 | | 1  2  3  4  5 | 0  3.06  13.28  23.30  33.32 | | 1.0  1.11  1.13  1.21  1.37 | | 0.99  1.00  1.06  1.18 | 1.25  1.26  1.39  1.58 | 506  637  775  421  315 | | | 60,701*  79,119  102,311  53,154  36,782 | | |  |
| Rayner | 2001-2013 | | 1  2  3  4 | 0.23  2.33  10.02  30.29 | | 0.94  0.90  0.95  1.00 (ref) | | 0.80  0.75  0.82 | 1.10  1.07  1.11 | 237  237  240  242 | | | 2186^†^  2185  2182  2180 | | |  |
| Romaguera | 1991-2007 | | 1  2  3  4 | 0  1.60  9.36  31.66 | | 1.0  0.97  1.04  1.06 | | 0.86  0.96  0.90 | 1.10  1.13  1.25 | 5837  1702  3425  720 | | | 2512^†^  332  340  406 | | |  |
| Scheffers | 1993-2010 | | 1  2  3  4  5 | 0  1.12  4.12  12.26  24.98 | | 1.0  1.00  0.98  0.97  0.98 | | 0.85  0.84  0.84  0.80 | 1.17  1.14  1.14  1.21 | 305  299  363  379  131 | | | 5364^†^  7576  9419  9182  3130 | | |  |

|  |  |  | |  |  | |  | |  | | |  | | |  | |  |  |  |
| --- | --- | --- | --- | --- | --- | --- | --- | --- | --- | --- | --- | --- | --- | --- | --- | --- | --- | --- | --- |
| First author | Study period | | Quantile | Exposure (g/day) | |  | | Ratio |  | | Cases | | | Non-cases or Person years* | | | | |  |
| F Studies used in the primary analysis of **sugar-sweetened** **beverages** | | | | | | | | | | | | | | | | |  |  |  |
|  |  | |  |  | | *Median* | | L95%CI | U95%CI |  | | | |  | | | | | |
| Bhupathiraju  (Men, Caffeinated) | 1986-2008 | | 1  2  3  4 | 0  2.73  14.04  39 | | 1.0  1.08  1.19  1.29 | | 1.01  1.09  1.14 | 1.15  1.28  1.47 | 3986  2039  1045  300 | | | 953,918*  440,607  175,261  45,128 | | |  | |  |  |
| Bhupathiraju  (Women, Caffeinated) | 1984-2008 | | 1  2  3  4 | 0  2.73  16.77  39 | | 1.0  1.05  1.19  1.33 | | 0.95  1.06  1.10 | 1.16  1.34  1.60 | 1186  897  431  472 | | | 328,351*  236,102  145,695  32,599 | | |  | |  |  |
| Bhupathiraju  (Women, Caffeine-free) | 1984-2008 | | 1  2  3  4 | 0  2.73  11.31  39 | | 1.0  1.02  1.14  1.37 | | 0.93  1.01  1.08 | 1.13  1.29  1.74 | 1197  1044  545  79 | | | 332,802*  273,819  120,909  15,208 | | |  | |  |  |
| Bhupathiraju (Men, Caffeine-free) | 1986-2008 | | 1  2  3  4 | 0  2.73  15.6  44.07 | | 1.0  1.01  1.02  1.20 | | 0.95  0.95  1.01 | 1.07  1.11  1.42 | 3705  2503  1008  154 | | | 869,023*  530,918  186,694  28,279 | | |  | |  |  |
| De Koning | 1986-2006 | | 1  2  3  4 | 0  2.56  11.11  36.11 | | 1.0  1.09  1.07  1.24 | | 0.97  0.95  1.09 | 1.22  1.20  1.40 | 586  629  685  780 | | | 167,462*  165,515  189,851  187,709 | | |  | |  |  |
| Eshak  (Men) | 1990-2000 | | 1  2  3  4 | 1.39  8.33  19.45  33.34 | | 1.0  0.86  0.83  0.98 | | 0.68  0.61  0.68 | 1.08  1.12  1.42 | 261  121  58  44 | | | 5,894^†^  3,205  1,539  1,015 | | |  | |  |  |
| Eshak  (Women) | 1990-2000 | | 1  2  3  4 | 1.39  8.33  19.45  33.34 | | 1.0  1.15  1.17  1.79 | | 0.88  0.78  1.11 | 1.51  1.76  2.89 | 200  83  30  27 | | | | 9,921^†^  3,325  1,168  694 | | | | | |
| Fagherazzi | 1993-2007 | | 1  2  3  4  5 | 0  0.72  1.96  4.03  7.24 | | 1.0  1.28  1.12  1.22  1.30 | | 1.06  0.86  0.94  1.02 | 1.55  1.45  1.57  1.66 | 1046  125  61  64  73 | | | 679,281*  56,559  34,329  34,230  33,696 | | |  | |  |  |
| Gardener | 1993-~2005 | | 1  2  3 | 0  11.13  65.23 | | 1.0  1.10  1.13 | | 0.86  0.81 | 1.40  1.61 | 154  160  54 | | | | 754^†^  670  227 | | | | | |
| Montonen | 1967-1979 | | 1  2  3  4 | 0  0.11  1.37  15.03 | | 1.0  0.78  0.97  1.61 | | 0.39  0.54  0.94 | 1.58  1.76  2.74 | 25  12  21  33 | | | 716^†^  446  552  555 | | |  | |  |  |
| O’Connor | 1993-2006 | | 1  2  3  4 | 0  3.68  8.73  24.60 | | 1.0  0.97  0.98  1.13 | | 0.80  0.80  0.94 | 1.18  1.19  1.36 | 418  130  135  164 | | | 11,425^†^  4167  4108  4106 | | |  | |  |  |
| Odegaard | 1993-2004 | | 1  2  3  4 | 0  1.85  3.70  19.26 | | 1.0  1.11  0.98  1.34 | | 0.97 0.81 1.17 | 1.26  1.29  1.52 | 1615  247  111  300 | | | 185,645*  25,285  13,104  25,140 | | |  | |  |  |
| Olsson | 1991-2016 | | 1  2  3  4 | 0  2.49  9.99  20.05 | | 1.0  1.03  1.06  1.06 | | 0.94  0.97  0.97 | 1.12  1.15  1.16 | 1746  749  723  828 | | | 221,228*  95,790  85,689  86,478 | | |  | |  |  |
| Palmer | 1995-2005 | | 1  2  3  4  5 | 0  4.49  22.22  39.00  55.78 | | 1.0  1.96  1.14  1.27  1.51 | | 0.87  1.02  1.12  1.31 | 1.06  1.27  1.47  1.75 | 733  783  656  280  261 | | | 96,266*  111,418  78,319  29,273  23,608 | | |  | |  |  |
| Papier  (Men) | 2005-2013 | | 1  2  3 | 1.28  19.45  58.34 | | 1.0  1.0  1.3 | | 0.8  0.9 | 1.2  2.1 | 236  168  33 | | | 8,624^†^  7,348  1,050 | | |  | |  |  |
| Papier  (Women) | 2005-2013 | | 1  2  3 | 1.28  19.45  58.34 | | 1.0  1.6  2.4 | | 1.2  1.5 | 2.1  3.9 | 142  88  28 | | | 13,149^†^  7,045  1,264 | | |  | |  |  |
| Paynter  (Men) | 1987-99 | | 1  2  3  4 | 2.6  26  39  65 | | 1.0  1.03  0.96  1.09 | | 0.80  0.80  0.89 | 1.35  1.15  1.33 | 331  67  182  138 | | | 2,226^†^  437  1,233  800 | | |  | |  |  |
| Paynter  (Women) | 1987-99 | | 1  2  3  4 | 2.6  26  39  65 | | 1.0  1.21  1.20  1.17 | | 0.97  1.00  0.94 | 1.51  1.44  1.46 | 320  103  182  114 | | | 3,190^†^  793  1,308  780 | | |  | |  |  |
| Ramne | 1991-2016 | | 1  2  3  4  5 | 0  6.06  14.63  24.56  35.87 | | 1.0  1.09  1.06  1.05  1.20 | | 0.93  0.79  0.78  0.88 | 1.29  1.42  1.40  1.60 | 330  263  53  53  51 | | | 1709^†^  1208  257  254  204 | | |  | |  |  |
| Romaguera | 1991-2007 | | 1  2  3  4 | 0  2.03  9.91  44.75 | | 1.0  1.19  1.07  1.29 | | 0.91  0.94  1.02 | 1.56  1.21  1.63 | 3948  964  1599  605 | | | 4401^†^  1070  2166  621 | | |  | |  |  |
| Sakurai | 2003-2010 | | 1  2  3  4 | 0  4.68  18.72  81.9 | | 1.0  0.97  1.11  1.34 | | 0.57  0.74  0.72 | 1.64  1.66  2.36 | 55  19  72  24 | | | 3554*  1494  4825  1381 | | |  | |  |  |
| Stern | 2006-2013 | | 1  2  3  4  5 | 1.28  2.62  6.52  16.71  33.43 | | 1.0  1.08  1.13  1.20  1.32 | | 0.95  1.00  1.05  1.17 | 1.22  1.28  1.37  1.49 | 417  558  763  501  916 | | | 25,700*  32,390  43,078  26,787  43,760 | | |  | |  |  |
| Torres-Ibarra | 2004-2018 | | 1  2  3 | 1.11  8.36  39.56 | | 1.0  1.0  1.5 | | 0.6  0.8 | 1.7  2.8 | 18  56  35 | | | 2372*  5113  2041 | | |  | |  |  |

|  |  |  | |  | |  | |  | |  | |  | | |  | |  |
| --- | --- | --- | --- | --- | --- | --- | --- | --- | --- | --- | --- | --- | --- | --- | --- | --- | --- |
| First author | Study period | | Quantile | | Exposure (g/day) | |  | | Ratio |  | Cases | | | Non-cases or Person years* | | | |
| G Studies used in the primary analysis of **glucose** | | | | | | | | | | | | | | | | |  |
|  |  | |  | |  | | *Median* | | L95%CI | U95%CI |  | | |  | | | |
| Ahmadi-Abhari | 1993-2006 | | 1  2  3  4  5 | | 7.95  12.73  16.45  19.82  25.36 | | 1.00  0.84  0.72  0.74  0.82 | | 0.66  0.56  0.57  0.62 | 1.11  0.94  0.98  1.10 | 200  161  138  132  122 | | | 662^†^  687  693  686  672 | | | |
| Kanehara  (Men) | 1990-2000, 1993-2003 | | 1  2  3  4 | | 7.59  12.60  16.61  22.55 | | 1.0  1.06  1.01  0.95 | | 0.85  0.80  0.74 | 1.32  1.28  1.22 | 180  181  170  159 | | 6769^†^  6768  6780  6790 | | |  |  |
| Kanehara (Women) | 1990-2000, 1993-2003 | | 1  2  3  4 | | 7.46  11.62  15.04  21.67 | | 1.0  0.84  0.95  1.23 | | 0.64  0.71  0.91 | 1.11  1.27  1.67 | 134  106  115  145 | | 9086^†^  9114  9105  9075 | | |  |  |
| Meyer | 1986-1992 | | 1  2  3  4  5 | | 6.95  15.75  19.4  23.5  28.1^y^ | | 1.00  0.95  1.11  1.18  1.30 | | 0.78  0.91  0.97  1.08 | 1.17  1.35  1.44  1.57 | 213  201  226  231  270 | | 39,958*  40,798  41,022  40,627  40,248 | | |  |  |
| Montonen | 1967-1979 | | 1  2  3  4 | | 5.6  10.6  15.9  27.5 | | 1.0  0.96  0.97  1.57 | | 0.61  0.62  1.04 | 1.50  1.53  2.37 | 41  38  37  59 | | | 1033^†^  1030  1032  1014 | | | |
| Schulze  (Men) | 1994-2005 | | 1  2  3  4  5 | | 6.6  11.1  15.0  20.2  31.4 | | 1.0  1.05  1.06  0.87  1.10 | | 0.79  0.79  0.63  0.81 | 1.40  1.42  1.19  1.50 | 106  99  96  77  113 | | 13,715*  13,541  13,555  13,577  13,397 | | |  |  |
| Schulze  (Women) | 1994-2005 | | 1  2  3  4  5 | | 9.6  13.1  15.4  18.1  24.3 | | 1.0  0.78  1.09  0.81  0.88 | | 0.52  0.75  0.53  0.58 | 1.15  1.60  1.22  1.33 | 62  62  100  69  62 | | 21,871*  21,740  21,963  21,609  21,497 | | |  |  |
|  |  | |  | |  | |  | |  |  |  | |  | | |  |  |

^†^Calculated as the number of total‐persons minus the number of cases.

^§^ All such, data not reported.

See Table S6 for references of studies.

# **Supplementary Table 8** Grade assessment for dose-response analysis of dietary sugar exposures and T2D incidence

| **No. of studies** | **Design** | **Risk of bias** | **Inconsistency** | **Indirectness** | **Imprecision** | **Other** | **Cases** | **Sample size** | **Pooled RR-effect size (95%CI)*** | **I^2^** | **P Value Hetero-geneity** | **Certainty (overall score)** |
| --- | --- | --- | --- | --- | --- | --- | --- | --- | --- | --- | --- | --- |
| **Dose-response relationship between sugar from SSB and T2D** | | | | | | | | | | | | |
| 17 | Observational studies | Serious^a^ | Not serious | Not serious | Not serious | none | 43532 | 541288 | 1.25 (1.17, 1.35) | 44.7% | 0.0114 | MODERATE |
| **Dose-response relationship between sugar from fruit juice and T2D** | | | | | | | | | | | | |
| 14 | Observational studies | Serious^a^ | Not serious | Not serious | Not serious | none | 43065 | 490413 | 1.05 (>1.00, 1.11) | 37.6% | 0.0704 | MODERATE |
| **Dose-response relationship between total sugars and T2D** | | | | | | | | | | | | |
| 4 | Observational studies | Very serious^a, b^ | Not serious | Not serious | Not serious | none | 13675 | 109858 | 0.96 (0.94, 0.98) | 0.3% | 0.4042 | LOW |
| **Dose-response relationship between added sugars and T2D** | | | | | | | | | | | | |
| 2 | Observational studies | Serious^a^ | Not serious | Not serious | Serious^e^ | none | 4796 | 31004 | 0.99 (0.96, 1.01) | 0.0% | 0.9085 | LOW |
| **Dose-response relationship between sucrose and T2D** | | | | | | | | | | | | |
| 7 | Observational studies | Serious^a^ | Not serious | Not serious | Not serious | none | 9065 | 223238 | 0.95 (0.91, <1.00) | 37.7% | 0.1176 | MODERATE |
| **Dose-response relationship between fructose and T2D** | | | | | | | | | | | | |
| 5 | Observational studies | Serious^a^ | Serious^c^ | Not serious | Serious^d^ | none | 4101 | 158136 | 0.98 (0.83, 1.15) | 75.5% | 0.0004 | VERY LOW |
| **Dose-response relationship between glucose and T2D** | | | | | | | | | | | | |
| 5 | Observational studies | Serious^a^ | Serious | Not serious | Serious^d^ | none | 4101 | 158136 | 1.01 (0.83, 1.23) | 62.4% | 0.0140 | VERY LOW |

* Pooled effect size for typical serving doses of SSB (39 g of sugar) and fruit juice (23.3 g of sugar) and effect sizes for 20-gram doses of total sugar, added sugar, sucrose, fructose and glucose.

Data deriving from cohort studies begin with a high grading (due to use of ROBINS-E) (121).

a. Downgraded by one level for risk of bias, since all studies were rated with a moderate risk of bias.

b. Downgraded by one level for very serious risk of bias, since one study was rated as a high risk of bias (Sluijs et al,.due to the case-cohort study design i.e., poor representativeness of cases).

c. Downgraded by one level for inconsistency since point estimates and 95% CI did not fully overlap between studies and I^2^ was substantial.

d. Downgraded by one level for imprecision since 95% CI crosses threshold of a minimal importance.

e. Downgraded by one level for imprecision, since studies come from different sections of the same cohort, limiting the diversity of evidence.

DR: A progressive dose–response relationship that was positive for SSB and fruit juice and inverse for sucrose intake, yet no upgrading was applied due to the residual confounding common to cohort studies.

# **Supplementary Table 9** **A-G** Results from leave-one-out sensitivity analyses for each sugar category. See Table S6 for references of studies.

## 9-A Total Sugar

| Excluded Study | Linear Model Slope | Lower 95% CI | Upper 95% CI | Slope change % | Absolute Difference | Standardized Difference | Slope falls within full CI |
| --- | --- | --- | --- | --- | --- | --- | --- |
| nan | -0.0019693 | -0.0031697 | -0.00076901 | 0 | 0 | 0 | True |
| Sluijs et al., 2013 | -0.0010442 | -0.0036675 | 0.0015792 | -46.98 | 0.00092519 | 1.5107 | True |
| Ahmadi-Abhari et al., 2014 | -0.0013468 | -0.0033552 | 0.00066169 | -31.613 | 0.00062258 | 1.0166 | True |
| Montonen et al. 2007 | -0.0020616 | -0.0032732 | -0.0008501 | 4.6859 | -9.2281E-05 | -0.15068 | True |
| Kanehara et al. 2022 - m | -0.0020119 | -0.0032677 | -0.00075607 | 2.1589 | -4.2516E-05 | -0.069423 | True |
| Kanehara et al. 2022 - f | -0.0021645 | -0.0033902 | -0.00093879 | 9.9102 | -0.00019517 | -0.31868 | True |

## 9-B Total Sucrose

| Excluded Study | Linear Model Slope | Lower 95% CI | Upper 95% CI | Slope change % | Absolute Difference | Standardized Difference | Slope falls within full CI |
| --- | --- | --- | --- | --- | --- | --- | --- |
| nan | -0.0023639 | -0.0045593 | -0.00016856 | 0 | 0 | 0 | True |
| Meyer et al.,2000 | -0.0018467 | -0.0041991 | 0.00050567 | -21.878 | 0.00051718 | 0.46173 | True |
| Janket et al., 2003 | -0.0019985 | -0.0043288 | 0.00033185 | -15.459 | 0.00036544 | 0.32626 | True |
| Schulze et al., 2008 m | -0.0018022 | -0.0040687 | 0.00046425 | -23.76 | 0.00056167 | 0.50146 | True |
| Schulze et al., 2008 w | -0.0024886 | -0.0047929 | -0.00018421 | 5.273 | -0.00012465 | -0.11129 | True |
| Ahmadi et al., 2014 | -0.002502 | -0.0049257 | -7.8229E-05 | 5.8397 | -0.00013805 | -0.12325 | True |
| Montonen et al. 2007 | -0.002549 | -0.0048304 | -0.00026756 | 7.8294 | -0.00018508 | -0.16524 | True |
| Olsson et al., 2021 | -0.0036477 | -0.0057329 | -0.0015625 | 54.308 | -0.0012838 | -1.1462 | True |
| Kanehara et al. 2022 - m | -0.0022592 | -0.004625 | 0.0001066 | -4.4289 | 0.00010469 | 0.093471 | True |
| Kanehara et al. 2022 - f | -0.0026636 | -0.0049031 | -0.00042417 | 12.679 | -0.00029972 | -0.26759 | True |

## 9-C Added Sugar

| Excluded Study | Linear Model Slope | Lower 95% CI | Upper 95% CI | Slope change % | Absolute Difference | Standardized Difference | Slope falls within full CI |
| --- | --- | --- | --- | --- | --- | --- | --- |
| nan | -0.00063604 | -0.0018159 | 0.00054382 | 0 | 0 | 0 | True |
| Olsson et al., 2021 | -0.00074321 | -0.0029183 | 0.0014319 | 16.848 | -0.00010716 | -0.17802 | True |
| Ramne et al., 2020 | -0.00059137 | -0.0019958 | 0.00081308 | -7.0242 | 4.4677E-05 | 0.074217 | True |

## 9-D SSB

| Excluded Study | Linear Model Slope | Lower 95% CI | Upper 95% CI | Slope change % | Absolute Difference | Standardized Difference | Slope falls within full CI |
| --- | --- | --- | --- | --- | --- | --- | --- |
| nan | 0.0058446 | 0.0040166 | 0.0076726 | 0 | 0 | 0 | True |
| Romaguera – Interact et al. 2013 | 0.0058032 | 0.0039355 | 0.007671 | -0.70781 | -4.1369E-05 | -0.044356 | True |
| Montonen et al. 2007 | 0.0057987 | 0.0039739 | 0.0076236 | -0.78495 | -4.5877E-05 | -0.04919 | True |
| Eshak et al., 2013, m | 0.0061124 | 0.0043131 | 0.0079117 | 4.5815 | 0.00026777 | 0.28711 | True |
| Eshak et al., 2013, f | 0.0057806 | 0.0039458 | 0.0076153 | -1.096 | -6.4057E-05 | -0.068683 | True |
| Fagherazzi et al, 2013 | 0.0057653 | 0.0039453 | 0.0075853 | -1.3574 | -7.9337E-05 | -0.085067 | True |
| Odegaard et al., 2010 | 0.0055297 | 0.0037596 | 0.0072998 | -5.3886 | -0.00031494 | -0.33769 | True |
| Palmer et al., 2008 | 0.0056621 | 0.0036957 | 0.0076284 | -3.123 | -0.00018253 | -0.19571 | True |
| De Koning et al., 2011 | 0.0059902 | 0.0039827 | 0.0079978 | 2.4919 | 0.00014564 | 0.15616 | True |
| Gardener et al., 2018 | 0.0058452 | 0.0039936 | 0.0076969 | 0.010816 | 6.3214E-07 | 0.00067779 | True |
| O’Connor et al., 2015 | 0.0059016 | 0.0039945 | 0.0078087 | 0.97552 | 5.7015E-05 | 0.061133 | True |
| Olsson et al., 2021 | 0.0060848 | 0.0041405 | 0.0080291 | 4.1101 | 0.00024022 | 0.25757 | True |
| Ramne et al., 2020 | 0.0059488 | 0.0040415 | 0.0078561 | 1.7829 | 0.0001042 | 0.11173 | True |
| Stern et al., 2019 | 0.0058165 | 0.0038154 | 0.0078176 | -0.48139 | -2.8135E-05 | -0.030167 | True |
| Torres-Ibarra et al., 2020 | 0.0058128 | 0.0039772 | 0.0076484 | -0.54425 | -3.1809E-05 | -0.034107 | True |
| Bhupathiraju 2013 - NHS - Caff | 0.0056882 | 0.0037311 | 0.0076454 | -2.6756 | -0.00015638 | -0.16767 | True |
| Bhupathiraju 2013 - NHS - Cafffree | 0.0061408 | 0.0042073 | 0.0080743 | 5.0683 | 0.00029622 | 0.31761 | True |
| Bhupathiraju 2013 - HPFS - Caff | 0.0053288 | 0.0036552 | 0.0070023 | -8.8262 | -0.00051585 | -0.55311 | True |
| Bhupathiraju 2013 - HPFS - Cafffree | 0.0057025 | 0.0038222 | 0.0075829 | -2.4308 | -0.00014207 | -0.15233 | True |
| Papier 2017 - m | 0.0059832 | 0.0040887 | 0.0078777 | 2.3709 | 0.00013857 | 0.14858 | True |
| Papier 2017 - f | 0.0057101 | 0.003892 | 0.0075282 | -2.3017 | -0.00013453 | -0.14424 | True |
| Paynter 2006 - m | 0.0062577 | 0.0045818 | 0.0079335 | 7.0673 | 0.00041305 | 0.44288 | True |
| Paynter 2006 - f | 0.0060231 | 0.0040471 | 0.0079992 | 3.0544 | 0.00017852 | 0.19141 | True |
| Sakurai et al 2014 | 0.0058329 | 0.00399 | 0.0076759 | -0.19957 | -1.1664E-05 | -0.012506 | True |

## 9-E Fruit Juice

| Excluded Study | Linear Model Slope | Lower 95% CI | Upper 95% CI | Slope Change % | Absolute Difference | Standardized Difference | Slope falls within full CI |
| --- | --- | --- | --- | --- | --- | --- | --- |
| nan | 0.0022087 | 6.9179E-05 | 0.0043483 | 0 | 0 | 0 | True |
| Romaguera – Interact et al. 2013 | 0.0021666 | -0.00019272 | 0.004526 | -1.9063 | -4.2105E-05 | -0.038571 | True |
| Montonen et al. 2007 | 0.0021746 | 3.5648E-05 | 0.0043136 | -1.5438 | -3.4098E-05 | -0.031237 | True |
| Mursu et al. 2014 | 0.0023775 | 0.00013262 | 0.0046224 | 7.6401 | 0.00016875 | 0.15459 | True |
| Eshak et al., 2013, m | 0.002304 | 0.00015182 | 0.0044563 | 4.3149 | 9.5305E-05 | 0.087306 | True |
| Eshak et al., 2013, f | 0.0022225 | 7.1434E-05 | 0.0043737 | 0.62526 | 1.381E-05 | 0.012651 | True |
| Bazzano, 2008 | 0.0014981 | -0.00057184 | 0.003568 | -32.176 | -0.00071068 | -0.65104 | True |
| Fagherazzi et al, 2013 | 0.0023194 | 8.5256E-05 | 0.0045535 | 5.0093 | 0.00011064 | 0.10136 | True |
| Odegaard et al., 2010 | 0.0020771 | -7.197E-05 | 0.0042262 | -5.9598 | -0.00013164 | -0.12059 | True |
| Palmer et al., 2008 | 0.0015947 | -0.00050595 | 0.0036953 | -27.801 | -0.00061405 | -0.56252 | True |
| Auerbach et al., 2017 | 0.0030775 | 0.00099799 | 0.005157 | 39.332 | 0.00086875 | 0.79584 | True |
| Bondonno et al., 2021 12 years | 0.0021991 | 3.8391E-05 | 0.0043599 | -0.43477 | -9.603E-06 | -0.0087971 | True |
| O’Connor et al., 2015 | 0.0022743 | 8.0852E-05 | 0.0044677 | 2.9664 | 6.5519E-05 | 0.060021 | True |
| Olsson et al., 2021 | 0.0023447 | -2.1811E-05 | 0.0047112 | 6.1555 | 0.00013596 | 0.12455 | True |
| Rayner et al. | 0.0021562 | -0.00022849 | 0.0045409 | -2.3778 | -5.2518E-05 | -0.048111 | True |
| Scheffers et al., 2020 | 0.0024786 | 0.00023093 | 0.0047263 | 12.218 | 0.00026986 | 0.24722 | True |

## 9-F Fructose

| Excluded Study | Linear Model Slope | Lower 95% CI | Upper 95% CI | Slope change % | Absolute Difference | Standardized Difference | Slope falls within full CI |
| --- | --- | --- | --- | --- | --- | --- | --- |
| nan | -0.0011412 | -0.0094992 | 0.0072168 | 0 | 0 | 0 | True |
| Meyer et al.,2000 | -0.0036148 | -0.011968 | 0.004738 | 216.75 | -0.0024736 | -0.58006 | True |
| Schulze et al., 2008 m | -0.00099232 | -0.011154 | 0.0091689 | -13.045 | 0.00014887 | 0.034912 | True |
| Schulze et al., 2008 w | -2.3091E-07 | -0.0095199 | 0.0095194 | -99.98 | 0.001141 | 0.26756 | True |
| Montonen et al. 2007 | -0.0024364 | -0.010949 | 0.006076 | 113.49 | -0.0012952 | -0.30372 | True |
| Ahmadi-Abhari et al., 2014 | 0.0020983 | -0.003571 | 0.0077676 | -283.87 | 0.0032395 | 0.75968 | True |
| Kanehara et al. 2022 - m | -0.00082564 | -0.010987 | 0.009336 | -27.652 | 0.00031556 | 0.074 | True |
| Kanehara et al. 2022 - f | -0.0020269 | -0.011614 | 0.0075607 | 77.608 | -0.00088566 | -0.20769 | True |

## 9-G Glucose

| Excluded Study | Linear Model Slope | Lower 95% CI | Upper 95% CI | Slope change % | Absolute Difference | Standardized Difference | Slope falls within full CI |
| --- | --- | --- | --- | --- | --- | --- | --- |
| nan | 0.00053944 | -0.009276 | 0.010355 | 0 | 0 | 0 | True |
| Schulze et al., 2008 - m | 0.00094267 | -0.011075 | 0.012961 | 74.75 | 0.00040323 | 0.08052 | True |
| Schulze et al., 2008 - w | 0.0018488 | -0.0089812 | 0.012679 | 242.73 | 0.0013094 | 0.26147 | True |
| Ahmadi-Abhari et al., 2014 | 0.0044311 | -0.0038502 | 0.012712 | 721.43 | 0.0038917 | 0.77711 | True |
| Montonen et al. 2007 | -0.0011236 | -0.011224 | 0.0089763 | -308.29 | -0.001663 | -0.33208 | True |
| Kanehara et al. 2022 - m | 0.0016043 | -0.010016 | 0.013225 | 197.4 | 0.0010649 | 0.21264 | True |
| Kanehara et al. 2022 - f | -0.00073676 | -0.011226 | 0.0097522 | -236.58 | -0.0012762 | -0.25484 | True |
| Meyer et al 2000 | -0.0031292 | -0.012571 | 0.0063127 | -680.08 | -0.0036686 | -0.73257 | True |

**Supplementary Figures 3** A-G Linear and cubic spline fits for all sugar types by study level and aggregated.


Panels depict dose-response relationships modeled using linear and non-linear approaches. Shaded regions represent the 95% confidence intervals (CIs). Red shaded regions correspond to the non-linear model fit, using restricted cubic splines to capture potential non-linearity in the relationship. Grey shaded regions represent the linear model fit, assuming a constant rate of change across all dose levels. Solid lines show the point estimates for each mode: solid red line reflects the non-linear model; dashed grey line corresponds to the linear model. The x-axis represent maximum intake ranges across all included studies for a given sugar type and the y-axis shows relative risk for T2D.

# **References**

1. Haddaway NR, Page MJ, Pritchard CC, McGuinness LA. PRISMA2020: An R package and Shiny app for producing PRISMA 2020‐compliant flow diagrams, with interactivity for optimised digital transparency and Open Synthesis. *Campbell systematic reviews*. 2022;18(2):e1230.

2. Alcada MN, Monteiro R, Calhau C, Azevedo I. Orange juice vs soft drinks. 2009.

3. Ashton L, Cade J, Burley V. A type 2 diabetes mellitus prevention index predicts incident diabetes in the UK Women's Cohort Study. *Proceedings of the Nutrition Society*. 2013;72(OCE4):E257.

4. Barclay AW, Flood VM, Rochtchina E, Mitchell P, Brand-Miller JC. Glycemic index, dietary fiber, and risk of type 2 diabetes in a cohort of older Australians. *Diabetes care*. 2007;30(11):2811-3.

5. Barrio-Lopez MT, Martinez-Gonzalez MA, Fernandez-Montero A, Beunza JJ, Zazpe I, Bes-Rastrollo M. Prospective study of changes in sugar-sweetened beverage consumption and the incidence of the metabolic syndrome and its components: the SUN cohort. *British Journal of Nutrition*. 2013;110(9):1722-31.

6. Bauer F, Beulens JW, Van Der A DL, Wijmenga C, Grobbee DE, Spijkerman AM et al. Dietary patterns and the risk of type 2 diabetes in overweight and obese individuals. *European journal of nutrition*. 2013;52:1127-34.

7. A. B. Sugar-sweetened beverages increase risk for type 2 diabetes in African-American women. *Journal of Clinical Outcomes Management*. 2008;15:427-9.

8. den Braver NR, Rutters F, van der Spek ALK, Ibi D, Looman M, Geelen A et al. Adherence to a food group-based dietary guideline and incidence of prediabetes and type 2 diabetes. *European journal of nutrition*. 2020;59:2159-69.

9. Chen Z, Khandpur N, Desjardins C, Wang L, Monteiro CA, Rossato SL et al. Ultra-processed food consumption and risk of type 2 diabetes: three large prospective US cohort studies. *Diabetes Care*. 2023;46(7):1335-44.

10. Colditz GA, Manson J, Stampfer MJ, Rosner B, Willett WC, Speizer FE. Diet and risk of clinical diabetes in women. *The American journal of clinical nutrition*. 1992;55(5):1018-23.

11. Conklin AI, Monsivais P, Khaw K-T, Wareham NJ, Forouhi NG. Dietary diversity, diet cost, and incidence of type 2 diabetes in the United Kingdom: a prospective cohort study. *PLoS medicine*. 2016;13(7):e1002085.

12. den Biggelaar LJ, Eussen SJ, Sep SJ, Mari A, Ferrannini E, van Dongen MC et al. Associations of dietary glucose, fructose, and sucrose with β-cell function, insulin sensitivity, and type 2 diabetes in the Maastricht study. *Nutrients*. 2017;9(4):380.

13. den Biggelaar LJ, Sep SJ, Mari A, Ferrannini E, van Dongen MC, Wijckmans NE et al. Association of artificially sweetened and sugar-sweetened soft drinks with β-cell function, insulin sensitivity, and type 2 diabetes: the Maastricht study. *European journal of nutrition*. 2020;59:1717-27.

14. Dow C, Balkau B, Bonnet F, Mancini F, Rajaobelina K, Shaw J et al. Strong adherence to dietary and lifestyle recommendations is associated with decreased type 2 diabetes risk in the AusDiab cohort study. *Preventive medicine*. 2019;123:208-16.

15. Dhingra R, Sullivan L, Jacques PF, Wang TJ, Fox CS, Meigs JB et al. Soft drink consumption and risk of developing cardiometabolic risk factors and the metabolic syndrome in middle-aged adults in the community. *Circulation*. 2007;116(5):480-8.

16. Drouin-Chartier J-P, Zheng Y, Li Y, Malik V, Pan A, Bhupathiraju SN et al. Changes in consumption of sugary beverages and artificially sweetened beverages and subsequent risk of type 2 diabetes: results from three large prospective US cohorts of women and men. *Diabetes care*. 2019;42(12):2181-9.

17. Duan M-J, Dekker LH, Carrero J-J, Navis G. Blood lipids-related dietary patterns derived from reduced rank regression are associated with incident type 2 diabetes. *Clinical Nutrition*. 2021;40(7):4712-9.

18. Duffey KJ, Gordon-Larsen P, Steffen LM, Jacobs Jr DR, Popkin BM. Drinking caloric beverages increases the risk of adverse cardiometabolic outcomes in the Coronary Artery Risk Development in Young Adults (CARDIA) Study. *The American journal of clinical nutrition*. 2010;92(4):954-9.

19. de ICkd. Adherence to predefined dietary patterns and incident type 2 diabetes in European populations: EPIC-InterAct Study. *Diabetologia*. 2014;57:321-33.

20. Ericson U, Brunkwall L, Alves Dias J, Drake I, Hellstrand S, Gullberg B et al. Food patterns in relation to weight change and incidence of type 2 diabetes, coronary events and stroke in the Malmö Diet and Cancer cohort. *European journal of nutrition*. 2019;58:1801-14.

21. Fantino M. Consumption of artificially and sugar-sweetened beverages and incident type 2 diabetes: methodologic concern about a recent epidemiological study. *The American journal of clinical nutrition*. 2013;98(1):246-58.

22. Ferreira-Pêgo C, Babio N, Bes-Rastrollo M, Corella D, Estruch R, Ros E et al. Frequent consumption of sugar-and artificially sweetened beverages and natural and bottled fruit juices is associated with an increased risk of metabolic syndrome in a Mediterranean population at high cardiovascular disease risk. *The Journal of nutrition*. 2016;146(8):1528-36.

23. Fresan U, Gea A, Bes-Rastrollo M, Basterra-Gortari F, Carlos S, Martinez-Gonzalez M. Substitution of water or fresh juice for bottled juice and type 2 diabetes incidence: The SUN cohort study. *Nutrition, Metabolism and Cardiovascular Diseases*. 2017;27(10):874-80.

24. Hayashino Y, Fukuhara S, Okamura T, Yamato H, Tanaka H, Tanaka T et al. A prospective study of passive smoking and risk of diabetes in a cohort of workers: the High-Risk and Population Strategy for Occupational Health Promotion (HIPOP-OHP) study. *Diabetes care*. 2008;31(4):732-4.

25. Hirahatake KM, Jacobs DR, Shikany JM, Jiang L, Wong ND, Odegaard AO. Cumulative average dietary pattern scores in young adulthood and risk of incident type 2 diabetes: the CARDIA study. *Diabetologia*. 2019;62:2233-44.

26. Hodge AM, English DR, O'Dea K, Giles GG. Dietary patterns and diabetes incidence in the Melbourne Collaborative Cohort Study. *American journal of epidemiology*. 2007;165(6):603-10.

27. Horikawa C, Yoshimura Y, Kamada C, Tanaka S, Tanaka S, Matsunaga S et al. Is the proportion of carbohydrate intake associated with the incidence of diabetes complications?—an analysis of the Japan Diabetes Complications Study. *Nutrients*. 2017;9(2):113.

28. Horikawa C, Yoshimura Y, Kamada C, Tanaka S, Tanaka S, Hanyu O et al., editors. Is Carbohydrate Intake Associated with the Incidence of Diabetes Complications? Japan Diabetes Complication Study (JDCS). DIABETES; 2016: AMER DIABETES ASSOC 1701 N BEAUREGARD ST, ALEXANDRIA, VA 22311-1717 USA.

29. Huang M, Quddus A, Stinson L, Shikany JM, Howard BV, Kutob RM et al. Artificially sweetened beverages, sugar-sweetened beverages, plain water, and incident diabetes mellitus in postmenopausal women: the prospective Women’s Health Initiative observational study. *The American journal of clinical nutrition*. 2017;106(2):614-22.

30. Imamura F, O’Connor L, Ye Z, Mursu J, Hayashino Y, Bhupathiraju SN et al. Consumption of sugar sweetened beverages, artificially sweetened beverages, and fruit juice and incidence of type 2 diabetes: systematic review, meta-analysis, and estimation of population attributable fraction. *Bmj*. 2015;351.

31. Jing Y, Han TS, Alkhalaf MM, Lean ME. Attenuation of the association between sugar-sweetened beverages and diabetes risk by adiposity adjustment: a secondary analysis of national health survey data. *European journal of nutrition*. 2019;58:1703-10.

32. Jo G KS, Cho Y, Shin MJ. . Soft drink consumption increases the risk of hypertension in Korean adults: a prospective cohort study. *Circulation*. 2017;135.

33. Khalangot MD, Kovtun VA, Gurianov VG, Pysarenko YM, Kravchenko VI. Evaluation of type 2 diabetes prevention through diet modification in people with impaired glucose regulation: a population-based study. *Primary Care Diabetes*. 2019;13(6):535-41.

34. Krishnan S, Coogan PF, Boggs DA, Rosenberg L, Palmer JR. Consumption of restaurant foods and incidence of type 2 diabetes in African American women. *The American Journal of Clinical Nutrition*. 2010;91(2):465-71.

35. Lang A, Kuss O, Filla T, Schlesinger S. Association between per capita sugar consumption and diabetes prevalence mediated by the body mass index: results of a global mediation analysis. *European journal of nutrition*. 2021;60:2121-9.

36. Li M, Li X, Zhao Y, Zhang L, Yang J, Zhou M et al. The burden of ischemic heart disease and type 2 diabetes mellitus attributable to diet high in sugar‐sweetened beverages in China: An analysis for the Global Burden of Disease Study 2017. *Journal of diabetes*. 2021;13(6):482-93.

37. Liu M, Liu C, Zhang Z, Zhou C, Li Q, He P et al. Quantity and variety of food groups consumption and the risk of diabetes in adults: a prospective cohort study. *Clinical Nutrition*. 2021;40(12):5710-7.

38. Löfvenborg JE, Andersson T, Carlsson P-O, Dorkhan M, Groop L, Martinell M et al. Sweetened beverage intake and risk of latent autoimmune diabetes in adults (LADA) and type 2 diabetes. *European journal of endocrinology*. 2016;175(6):605-14.

39. López GE, Batis C, González C, Chávez M, Cortés-Valencia A, López-Ridaura R et al. EAT-Lancet Healthy Reference Diet score and diabetes incidence in a cohort of Mexican women. *European journal of clinical nutrition*. 2023;77(3):348-55.

40. Mekonnen TA, Odden MC, Coxson PG, Guzman D, Lightwood J, Wang YC et al. Health benefits of reducing sugar-sweetened beverage intake in high risk populations of California: results from the cardiovascular disease (CVD) policy model. *PloS one*. 2013;8(12):e81723.

41. Mohan V, Radhika G, Sathya RM, Tamil SR, Ganesan A, Sudha V. Dietary carbohydrates, glycaemic load, food groups and newly detected type 2 diabetes among urban Asian Indian population in Chennai, India (Chennai Urban Rural Epidemiology Study 59). *British journal of nutrition*. 2009;102(10):1498-506.

42. Muraki I, Imamura F, Manson JE, Hu FB, Willett WC, van Dam RM et al. Fruit consumption and risk of type 2 diabetes: results from three prospective longitudinal cohort studies. *Bmj*. 2013;347.

43. Muraki I, Imamura F, Hu FB, Willett WC, van Dam R, Sun Q. Abstract MP94: Consumption of Specific Fruits and Incidence of Type 2 Diabetes in Men and Women. Am Heart Assoc; 2013.

44. Murray I, Kazman S. Sugar-sweetened beverages, weight gain, and diabetes. *JAMA*. 2005;293(4):422-3.

45. Naja F, Hwalla N, Itani L, Salem M, Azar ST, Zeidan MN et al. Dietary patterns and odds of Type 2 diabetes in Beirut, Lebanon: a case–control study. *Nutrition & metabolism*. 2012;9:1-11.

46. Nettleton JA, Lutsey PL, Wang Y, Lima JA, Michos ED, Jacobs Jr DR. Diet soda intake and risk of incident metabolic syndrome and type 2 diabetes in the Multi-Ethnic Study of Atherosclerosis (MESA). *Diabetes care*. 2009;32(4):688-94.

47. Nettleton JA, Steffen LM, Ni H, Liu K, Jacobs Jr DR. Dietary patterns and risk of incident type 2 diabetes in the Multi-Ethnic Study of Atherosclerosis (MESA). *Diabetes care*. 2008;31(9):1777-82.

48. O'Connor L, Imamura F, Lentjes M, Khaw K-T, Wareham N, Forouhi N, editors. Sweet beverage intake and type 2 diabetes. ANNALS OF NUTRITION AND METABOLISM; 2015: KARGER ALLSCHWILERSTRASSE 10, CH-4009 BASEL, SWITZERLAND.

49. O'Neill K, Fitzgerald A, Kearney P, editors. Impact of sugar sweetened beverages on incidence of type 2 diabetes in Ireland. DIABETOLOGIA; 2017: SPRINGER 233 SPRING ST, NEW YORK, NY 10013 USA.

50. O'Neill KN, Fitzgerald AP, Kearney PM. Impact of population distribution shifts in sugar-sweetened beverage consumption on type II diabetes incidence in Ireland. *Annals of Epidemiology*. 2020;41:1-6.

51. Odegaard AO, Koh WP, Juan J-M, Gross MD, Pereira MA, editors. Risk of Incident Type 2 Diabetes According to Frequency of Western-tyle Fast Food Intake in an Eastern Country. DIABETES; 2011: AMER DIABETES ASSOC 1701 N BEAUREGARD ST, ALEXANDRIA, VA 22311-1717 USA.

52. Olofsson C, Discacciati A, Åkesson A, Orsini N, Brismar K, Wolk A. Changes in fruit, vegetable and juice consumption after the diagnosis of type 2 diabetes: a prospective study in men. *British Journal of Nutrition*. 2017;117(5):712-9.

53. Osei TB, van Dijk A-M, Dingerink S, Chilunga FP, Beune E, Meeks KAC et al. Reduced RANK Regression-Derived dietary patterns related to the fatty liver index and associations with type 2 diabetes mellitus among Ghanaian populations under transition: the RODAM study. *Nutrients*. 2021;13(11):3679.

54. Pan A, Malik V, Willett WC, Hu FB. Plain Water and Total Beverage Intakes and Risk of Type 2 Diabetes in Young and Middle-aged Women. Am Heart Assoc; 2012.

55. Palmer JR, Boggs DA, Krishnan S, Hu FB, Singer M, Rosenberg L. Sugar-sweetened beverages and incidence of type 2 diabetes mellitus in African American women. *Archives of internal medicine*. 2008;168(14):1487-92.

56. Pankow J, Lutsey P, Selvin E, Huxley R, Couper D, Mosley T et al. 20-YEAR RISK OF TYPE 2 DIABETES IN RELATION TO MODIFIABLE RISK FACTORS: THE ARIC STUDY. 2011.

57. Papakonstantinou E, Panagiotakos DB, Pitsavos C, Chrysohoou C, Zampelas A, Skoumas Y et al. Food group consumption and glycemic control in people with and without type 2 diabetes: the ATTICA study. *Diabetes care*. 2005;28(10):2539-40.

58. Parnell LD, Noel SE, Bhupathiraju SN, Smith CE, Haslam DE, Zhang X et al. Metabolite patterns link diet, obesity, and type 2 diabetes in a Hispanic population. *Metabolomics*. 2021;17:1-12.

59. Pereira MA, Parker ED, Folsom AR. Intake of sugar sweetened beverages, fruit juice, and incidence of type 2 diabetes: A prospective study of postmenopausal women. *Diabetes*. 2005;54:A258.

60. Perez-Heras A RE, Serra-Mir M, Vinyas C, Mestre C, Alegret M, et al. . Intake of simple sugar-S from sweetened beverages is associated with cancer incidence and mortality in the predimed study cohort. . *Revista espanola de nutricion humana y dietetica*. 2016;20:425‐6.

61. Pomares-Millan H, Atabaki-Pasdar N, Coral D, Johansson I, Giordano GN, Franks PW. Estimating the Direct Effect between Dietary Macronutrients and Cardiometabolic Disease, Accounting for Mediation by Adiposity and Physical Activity. *Nutrients*. 2022 Mar 13;14(6).

62. Qi L, Cornelis MC, Zhang C, Van Dam RM, Hu FB. Genetic predisposition, Western dietary pattern, and the risk of type 2 diabetes in men. *The American journal of clinical nutrition*. 2009;89(5):1453-8.

63. Ramne S, Dias JA, González-Padilla E, Olsson K, Lindahl B, Engström G et al. Association between added sugar intake and mortality is nonlinear and dependent on sugar source in 2 Swedish population–based prospective cohorts. *The American journal of clinical nutrition*. 2019;109(2):411-23.

64. Rhee JJ, Mattei J, Hughes MD, Hu FB, Willett WC. Dietary diabetes risk reduction score, race and ethnicity, and risk of type 2 diabetes in women. *Diabetes care*. 2015;38(4):596-603.

65. Sahyoun NR, Anderson AL, Tylavsky FA, Lee JS, Sellmeyer DE, Harris TB. Dietary glycemic index and glycemic load and the risk of type 2 diabetes in older adults. *Am J Clin Nutr*. 2008 Jan;87(1):126-31.

66. Shams-White MM, Tjaden AH, Edelstein SL, Bassiouni S, Kahle LL, Kim C et al. The 2018 World Cancer Research Fund (WCRF)/American Institute for Cancer Research (AICR) score and diabetes risk in the Diabetes Prevention Program Outcomes Study (DPPOS). *BMC nutrition*. 2022;8(1):105.

67. Scheffers FR, Boer JM, Wijga AH, van der Schouw YT, Smit HA, Verschuren WM. Substitution of pure fruit juice for fruit and sugar-sweetened beverages and cardiometabolic risk in European Prospective Investigation into Cancer and Nutrition (EPIC)-NL: a prospective cohort study. *Public Health Nutr*. 2022 Jun;25(6):1504-14.

68. Schulze MB, Manson JE, Ludwig DS, Colditz GA, Stampfer MJ, Willett WC et al. Sugar-sweetened beverages, weight gain, and incidence of type 2 diabetes in young and middle-aged women. *Jama*. 2004 Aug 25;292(8):927-34.

69. Schulze M, Willett W, Manson J, Ludwig D, Colditz G, Stampfer M et al., editors. Consumption of sugar-sweetened soft drinks is related to greater weight gain and increased risk of type 2 diabetes in women. DIABETES; 2004: AMER DIABETES ASSOC 1701 N BEAUREGARD ST, ALEXANDRIA, VA 22311-1717 USA.

70. Schwingshackl L, Knüppel S, Michels N, Schwedhelm C, Hoffmann G, Iqbal K et al. Intake of 12 food groups and disability-adjusted life years from coronary heart disease, stroke, type 2 diabetes, and colorectal cancer in 16 European countries. *Eur J Epidemiol*. 2019 Aug;34(8):765-75.

71. Seino Y, Iizuka K, Suzuki A. Eating whole fruit, not drinking fruit juice, may reduce the risk of type 2 diabetes mellitus. *J Diabetes Investig*. 2021 Oct;12(10):1759-61.

72. Soto-Estrada G, Moreno Altamirano L, García-García JJ, Ochoa Moreno I, Silberman M. Trends in frequency of type 2 diabetes in Mexico and its relationship to dietary patterns and contextual factors. *Gaceta sanitaria*. 2018;32:283-90.

73. Srour B, Fezeu LK, Kesse-Guyot E, Allès B, Debras C, Druesne-Pecollo N et al. Ultraprocessed Food Consumption and Risk of Type 2 Diabetes Among Participants of the NutriNet-Santé Prospective Cohort. *JAMA Intern Med*. 2020 Feb 1;180(2):283-91.

74. Sugihiro T, Yoneda M, Ohno H, Oki K, Hattori N. Associations of nutrient intakes with obesity and diabetes mellitus in the longitudinal medical surveys of Japanese Americans. *J Diabetes Investig*. 2019 Sep;10(5):1229-36.

75. Takeuchi M, Horikawa C, Hatta M, Takeda Y, Nedachi R, Ikeda I et al. Secular Trends in Dietary Intake over a 20-Year Period in People with Type 2 Diabetes in Japan: A Comparative Study of Two Nationwide Registries; Japan Diabetes Complications Study (JDCS) and Japan Diabetes Clinical Data Management Study (JDDM). *Nutrients*. 2021 Sep 28;13(10).

76. Tasevska N, Pettinger M, Kipnis V, Midthune D, Tinker LF, Potischman N et al. Associations of Biomarker-Calibrated Intake of Total Sugars With the Risk of Type 2 Diabetes and Cardiovascular Disease in the Women's Health Initiative Observational Study. *Am J Epidemiol*. 2018 Oct 1;187(10):2126-35.

77. Teshima N, Shimo M, Miyazawa K, Konegawa S, Matsumoto A, Onishi Y et al. Effects of sugar-sweetened beverage intake on the development of type 2 diabetes mellitus in subjects with impaired glucose tolerance: the Mihama diabetes prevention study. *J Nutr Sci Vitaminol (Tokyo)*. 2015;61(1):14-9.

78. Tsilas CS, de Souza RJ, Mejia SB, Mirrahimi A, Cozma AI, Jayalath VH et al. Relation of total sugars, fructose and sucrose with incident type 2 diabetes: a systematic review and meta-analysis of prospective cohort studies. *Cmaj*. 2017;189(20):E711-E20.

79. van Dam RM, Rimm EB, Willett WC, Stampfer MJ, Hu FB. Dietary patterns and risk for type 2 diabetes mellitus in US men. *Annals of internal medicine*. 2002;136(3):201-9.

80. van 't Riet E, Dekker JM, Sun Q, Nijpels G, Hu FB, van Dam RM. Role of adiposity and lifestyle in the relationship between family history of diabetes and 20-year incidence of type 2 diabetes in U.S. women. *Diabetes Care*. 2010 Apr;33(4):763-7.

81. Viana Dias JP, Pimenta AM, de Souza Costa Sobrinho P, Miranda Hermsdorff HH, Bressan J, Nobre LN. Consumption of sweetened beverages is associated with the incidence of type 2 diabetes in Brazilian adults (CUME project). *Nutrition, Metabolism and Cardiovascular Diseases*. 2023;33(4):789-96.

82. Villegas R, Shu XO, Li H, Cai H, Gong Y, Gao Y-T et al., editors. Dietary patterns and the incidence of type 2 diabetes: The shanghai women health study. Diabetes; 2009: AMER DIABETES ASSOC 1701 N BEAUREGARD ST, ALEXANDRIA, VA 22311-1717 USA.

83. Vitale M, Masulli M, Cocozza S, Anichini R, Babini AC, Boemi M et al. Sex differences in food choices, adherence to dietary recommendations and plasma lipid profile in type 2 diabetes - The TOSCA.IT study. *Nutr Metab Cardiovasc Dis*. 2016 Oct;26(10):879-85.

84. von Ruesten A, Feller S, Bergmann MM, Boeing H. Diet and risk of chronic diseases: results from the first 8 years of follow-up in the EPIC-Potsdam study. *Eur J Clin Nutr*. 2013 Apr;67(4):412-9.

85. Voortman T, Kiefte-de Jong JC, Ikram MA, Stricker BH, van Rooij FJA, Lahousse L et al. Adherence to the 2015 Dutch dietary guidelines and risk of non-communicable diseases and mortality in the Rotterdam Study. *Eur J Epidemiol*. 2017 Nov;32(11):993-1005.

86. Wang M, Yu M, Fang L, Hu RY. Association between sugar‐sweetened beverages and type 2 diabetes: a meta‐analysis. *Journal of diabetes investigation*. 2015;6(3):360-6.

87. Welsh J, Dietz W. Sugar-sweetened beverage consumption is associated with weight gain and incidence of type 2 diabetes. *Clinical Diabetes*. 2005;23(4):150-2.

88. Xi B, Li S, Liu Z, Tian H, Yin X, Huai P et al. Intake of fruit juice and incidence of type 2 diabetes: a systematic review and meta-analysis. *PloS one*. 2014;9(3):e93471.

89. Yang X, Li Y, Wang C, Mao Z, Chen Y, Ren P et al. Association of plant-based diet and type 2 diabetes mellitus in Chinese rural adults: The Henan Rural Cohort Study. *J Diabetes Investig*. 2021 Sep;12(9):1569-76.

90. Yashpal S, Liese AD, Boucher BA, Wagenknecht LE, Haffner SM, Johnston LW et al. Metabolomic profiling of the Dietary Approaches to Stop Hypertension diet provides novel insights for the nutritional epidemiology of type 2 diabetes mellitus. *British journal of Nutrition*. 2022;128(3):487-97.

91. Zong G, Eisenberg DM, Hu FB, Sun Q. Consumption of Meals Prepared at Home and Risk of Type 2 Diabetes: An Analysis of Two Prospective Cohort Studies. *PLoS Med*. 2016 Jul;13(7):e1002052.

92. Ahmadi-Abhari S, Luben RN, Powell N, Bhaniani A, Chowdhury R, Wareham NJ et al. Dietary intake of carbohydrates and risk of type 2 diabetes: The European Prospective Investigation into Cancer-Norfolk study. *British journal of nutrition*. 2014;111(2):342-52.

93. Auerbach BJ. Cardiovascular Health Effects of 100% Fruit Juice Versus Whole Fruit in Postmenopausal Women: Results from the Women’s Health Initiative 2016.

94. Bazzano LA, Li TY, Joshipura KJ, Hu FB. Intake of fruit, vegetables, and fruit juices and risk of diabetes in women. *Diabetes care*. 2008;31(7):1311-7.

95. Bhupathiraju SN, Pan A, Malik VS, Manson JE, Willett WC, van Dam RM et al. Caffeinated and caffeine-free beverages and risk of type 2 diabetes. *The American journal of clinical nutrition*. 2013;97(1):155-66.

96. Bondonno NP, Davey RJ, Murray K, Radavelli-Bagatini S, Bondonno CP, Blekkenhorst LC et al. Associations between fruit intake and risk of diabetes in the AusDiab cohort. *The Journal of Clinical Endocrinology & Metabolism*. 2021;106(10):e4097-e108.

97. De Koning L, Malik VS, Rimm EB, Willett WC, Hu FB. Sugar-sweetened and artificially sweetened beverage consumption and risk of type 2 diabetes in men. *The American journal of clinical nutrition*. 2011;93(6):1321-8.

98. Eshak ES, Iso H, Mizoue T, Inoue M, Noda M, Tsugane S. Soft drink, 100% fruit juice, and vegetable juice intakes and risk of diabetes mellitus. *Clinical nutrition*. 2013;32(2):300-8.

99. Fagherazzi G, Vilier A, Sartorelli DS, Lajous M, Balkau B, Clavel-Chapelon F. Consumption of artificially and sugar-sweetened beverages and incident type 2 diabetes in the Etude Epidemiologique aupres des femmes de la Mutuelle Generale de l’Education Nationale–European Prospective Investigation into Cancer and Nutrition cohort. *The American journal of clinical nutrition*. 2013;97(3):517-23.

100. Gardener H, Moon YP, Rundek T, Elkind MS, Sacco RL. Diet soda and sugar-sweetened soda consumption in relation to incident diabetes in the Northern Manhattan Study. *Current developments in nutrition*. 2018;2(5):nzy008.

101. Janket S-J, Manson JE, Sesso H, Buring JE, Liu S. A prospective study of sugar intake and risk of type 2 diabetes in women. *Diabetes care*. 2003;26(4):1008-15.

102. Kanehara R, Goto A, Sawada N, Mizoue T, Noda M, Hida A et al. Association between sugar and starch intakes and type 2 diabetes risk in middle-aged adults in a prospective cohort study. *European Journal of Clinical Nutrition*. 2022;76(5):746-55.

103. Meyer KA, Kushi LH, Jacobs Jr DR, Slavin J, Sellers TA, Folsom AR. Carbohydrates, dietary fiber, and incident type 2 diabetes in older women. *The American journal of clinical nutrition*. 2000;71(4):921-30.

104. Montonen J, Järvinen R, Knekt P, Heliövaara M, Reunanen A. Consumption of sweetened beverages and intakes of fructose and glucose predict type 2 diabetes occurrence. *The Journal of nutrition*. 2007;137(6):1447-54.

105. Mursu J, Virtanen JK, Tuomainen T-P, Nurmi T, Voutilainen S. Intake of fruit, berries, and vegetables and risk of type 2 diabetes in Finnish men: the Kuopio Ischaemic Heart Disease Risk Factor Study. *The American journal of clinical nutrition*. 2014;99(2):328-33.

106. O'Connor L, Imamura F, Lentjes MA, Khaw KT, Wareham NJ, Forouhi NG. Prospective associations and population impact of sweet beverage intake and type 2 diabetes, and effects of substitutions with alternative beverages. *Diabetologia*. 2015 Jul;58(7):1474-83.

107. Odegaard AO, Koh W-P, Arakawa K, Yu MC, Pereira MA. Soft drink and juice consumption and risk of physician-diagnosed incident type 2 diabetes: the Singapore Chinese Health Study. *American journal of epidemiology*. 2010;171(6):701-8.

108. Olsson K, Ramne S, González-Padilla E, Ericson U, Sonestedt E. Associations of carbohydrates and carbohydrate-rich foods with incidence of type 2 diabetes. *British Journal of Nutrition*. 2021;126(7):1065-75.

109. Papier K, D'Este C, Bain C, Banwell C, Seubsman S-a, Sleigh A et al. Consumption of sugar-sweetened beverages and type 2 diabetes incidence in Thai adults: results from an 8-year prospective study. *Nutrition & diabetes*. 2017;7(6):e283-e.

110. Paynter NP, Yeh HC, Voutilainen S, Schmidt MI, Heiss G, Folsom AR et al. Coffee and sweetened beverage consumption and the risk of type 2 diabetes mellitus: the atherosclerosis risk in communities study. *Am J Epidemiol*. 2006 Dec 1;164(11):1075-84.

111. Ramne S, Drake I, Ericson U, Nilsson J, Orho-Melander M, Engström G et al. Identification of Inflammatory and Disease-Associated Plasma Proteins that Associate with Intake of Added Sugar and Sugar-Sweetened Beverages and Their Role in Type 2 Diabetes Risk. *Nutrients*. 2020 Oct 14;12(10).

112. Rayner J, D'Arcy E, Ross LJ, Hodge A, Schoenaker D. Carbohydrate restriction in midlife is associated with higher risk of type 2 diabetes among Australian women: A cohort study. *Nutr Metab Cardiovasc Dis*. 2020 Mar 9;30(3):400-9.

113. Romaguera D, Norat T, Wark PA, Vergnaud AC, Schulze MB, van Woudenbergh GJ et al. Consumption of sweet beverages and type 2 diabetes incidence in European adults: results from EPIC-InterAct. *Diabetologia*. 2013 Jul;56(7):1520-30.

114. Sakurai M, Nakamura K, Miura K, Takamura T, Yoshita K, Nagasawa SY et al. Sugar-sweetened beverage and diet soda consumption and the 7-year risk for type 2 diabetes mellitus in middle-aged Japanese men. *Eur J Nutr*. 2014 Feb;53(1):251-8.

115. Scheffers FR, Wijga AH, Verschuren WMM, van der Schouw YT, Sluijs I, Smit HA et al. Pure Fruit Juice and Fruit Consumption Are Not Associated with Incidence of Type 2 Diabetes after Adjustment for Overall Dietary Quality in the European Prospective Investigation into Cancer and Nutrition-Netherlands (EPIC-NL) Study. *J Nutr*. 2020 Jun 1;150(6):1470-7.

116. Schulze MB, Schulz M, Heidemann C, Schienkiewitz A, Hoffmann K, Boeing H. Carbohydrate intake and incidence of type 2 diabetes in the European Prospective Investigation into Cancer and Nutrition (EPIC)-Potsdam Study. *British journal of nutrition*. 2008;99(5):1107-16.

117. Sluijs I, Beulens JW, van der Schouw YT, van der AD, Buckland G, Kuijsten A et al. Dietary glycemic index, glycemic load, and digestible carbohydrate intake are not associated with risk of type 2 diabetes in eight European countries. *J Nutr*. 2013 Jan;143(1):93-9.

118. Stern D, Mazariegos M, Ortiz-Panozo E, Campos H, Malik VS, Lajous M et al. Sugar-sweetened soda consumption increases diabetes risk among Mexican women. *The Journal of nutrition*. 2019;149(5):795-803.

119. Torres-Ibarra L, Rivera-Paredez B, Hernández-López R, Canto-Osorio F, Sánchez-Romero LM, López-Olmedo N et al. Regular consumption of soft drinks is associated with type 2 diabetes incidence in Mexican adults: findings from a prospective cohort study. *Nutr J*. 2020 Nov 20;19(1):126.

120. O’Connor L, Imamura F, Lentjes MA, Khaw K-T, Wareham NJ, Forouhi NG. Prospective associations and population impact of sweet beverage intake and type 2 diabetes, and effects of substitutions with alternative beverages. *Diabetologia*. 2015;58:1474-83.
